# Recyclable fluorous cinchona alkaloid ester as a chiral promoter for asymmetric fluorination of β-ketoesters

**DOI:** 10.3762/bjoc.8.138

**Published:** 2012-08-03

**Authors:** Wen-Bin Yi, Xin Huang, Zijuan Zhang, Dian-Rong Zhu, Chun Cai, Wei Zhang

**Affiliations:** 1 School of Chemical Engineering, Nanjing University of Science and Technology, Xiao Ling Wei Street, Nanjing 210094, People’s Republic of China; 2 Department of Chemistry, University of Massachusetts Boston, 100 Morrissey Boulevard, Boston, MA 02125, USA

**Keywords:** asymmetric fluorination, β-ketoester, fluorous cinchona ester, organocatalysis, recyclable chiral promoter

## Abstract

A fluorous cinchona alkaloid ester has been developed as a chiral promoter for the asymmetric fluorination of β-ketoesters. It has comparable reactivity and selectivity to the nonfluorous versions of cinchona alkaloids and can be easily recovered from the reaction mixture by simple fluorous solid-phase extraction (F-SPE) and used for the next round of reaction without further purification.

## Introduction

Fluorinated organic compounds have unique properties because fluorine forms a strong carbon–fluorine bond with a small covalent radius and high electronegativity. Other than fluorinated polymers in materials science, organofluorine compounds have gained increasing popularity in medical chemistry and agricultural chemistry. Introducing one or a few fluorine atoms to biologically interesting molecules can significantly change the physical, chemical and biological properties [1–2]. The significant amount of publications on fluorinated small molecules, amino acids, carbohydrates, steroids and nucleosides indicates that organofluorine chemistry plays an important role in the life sciences [3–4].

**Figure 1:**
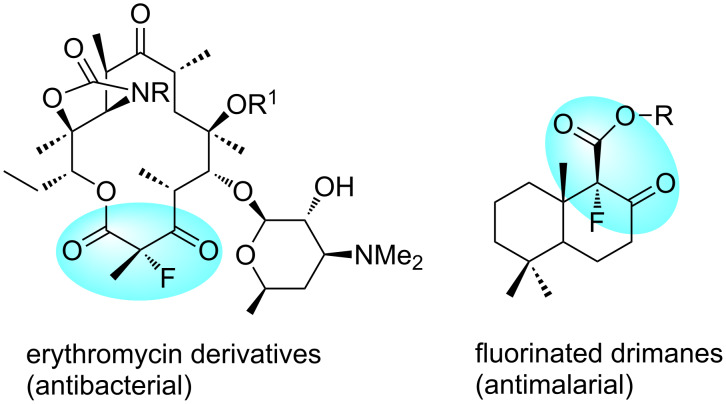
Biologically interesting α-fluorinated β-ketoesters.

A fluorine atom has been introduced to the α-position of some biologically interesting β-ketoesters, such as erythromycin and sesquiterpenic drimane (Figure 1) [5–6]. The achiral fluorination of β-ketoesters can be achieved by electrophilic reaction with Selectfluor (F-TEDA-BF_4_, 1-chloromethyl-4-fluoro-1,4-diazoniabicyclo[2.2.2]octane bis(tetrafluoroborate)), as developed by Bank [7–9]. The Cahard [10–12] and Shibata [13–14] groups combined cinchona alkaloids and Selectfluor for asymmetric fluorination of substrates such as imido-protected phenylglycines (up to 94% ee), indanones and tetralones (up to 91% ee), ethyl α-cyanotolyl acetates (up to 87% ee), and cyclic β-ketoesters (up to 80% ee) [15]. A catalytic approach for the cinchona alkaloids and Selectfluor combinations has also been developed [16]. The Togni group employed chiral titanium Lewis acid TiCl_2_(TADDOLate) for the asymmetric fluorination of β-ketoesters (up to 96% ee) [17–20]. Most Selectfluor-promoted asymmetric fluorinations require a stoichiometric amount of chiral promoters to suppress the competitively direct achiral fluorination. Different supported cinchona alkaloids have been developed as recyclable chiral promoters or organocatalysts. Among them, the Cahard group developed soluble polymer- and ionic-liquid-supported cinchona alkaloids for electrophilic fluorination [21–22]. The Fache and Soόs groups developed fluorous tag-attached cinchona alkaloids for catalytic Diels–Alder reactions [23–24]. Introduced in this paper is a new fluorous cinchona alkaloid ester for flourination of β-ketoesters. It is part of our recent effort on the development of recyclable fluorous reagents and organocatalysts for asymmetric synthesis [25–27].

## Results and Discussion

**Scheme 1:**
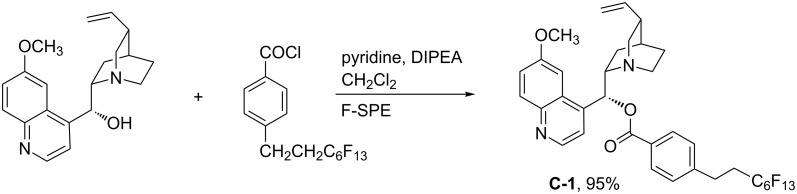
Preparation of quinine ester **C-1**.

Cinchona alkaloids and their derivatives have been well-explored in asymmetric synthesis [28]. We envisioned that the introduction of a fluorous tag could facilitate the recycling of cinchona alkaloids. The synthesis of fluorous quinine ester **C-1** was accomplished by the reaction of quinine with a fluorous acid chloride (Scheme 1). This compound was easily purified by fluorous-solid phase extraction (F-SPE) with a cartridge charged with fluorous silica gels [29–30]. It is stable in air and soluble in solvents such as CH_2_Cl_2_, CH_3_OH, and CH_3_CN.

**Figure 2:**
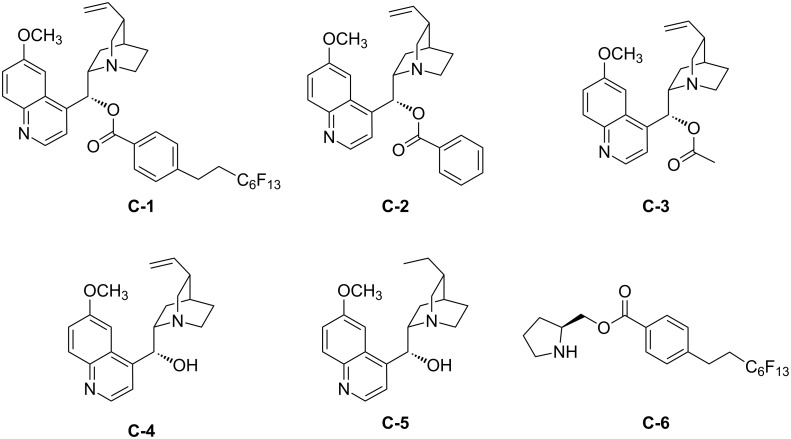
Promoters for asymmetric fluorination.

With the fluorous quinine ester **C-1** in hand, we explored the fluorination reaction using ethyl 2-methyl-3-oxo-3-phenylpropanoate (**1a**) as a model compound. Nonfluorous quinine esters, such as **C-2** and **C-3**, cinchona alkaloids **C-4** and **C-5**, and fluorous pyrrolidine ester **C-6**, were also evaluated (Figure 2). The results of the fluorination of β-ketoester **1a** with Selectfluor and different promoters are listed in Table 1. It was found that using MeCN as a solvent with 1 equiv of **C-1** gave fluorinated product **2a** in 49% yield and 65% ee (Table 1, entry 1). Compared to other promoters (Table 1, entries 2–5), **C-1** gave fluorinated products in a slightly low yield but better enantioselectivity. This may be attributed to the stereo and the electronic effect of the fluorous tag. Fluorous pyrrolidine **C-6** (Table 1, entry 6) gave the lowest product yield and ee among all six promoters. Reducing the amount of **C-1** from 1 equiv to 0.5 and 0.2 equiv significantly reduced the ee of the product (Table 1, entries 7 and 8). A control reaction without **C-1** gave **2a** in 35% yield as a racemic product (Table 1, entry 9). The results suggest that a stoichiometric amount of **C-1** is required to minimize the formation of achiral fluorination product by direct fluorination. Solvent screening indicated that using 1:1 CH_3_CN/CH_2_Cl_2_ gave product **2a** in 51% yield and 70% ee (Table 1, entry 15), which is better than using CH_3_CN alone. Other single or binary solvent systems containing toluene, THF, H_2_O, and CF_3_C_6_H_5_ did not afford better results (Table 1, entries 10–14). It was also found that lowering of the reaction temperature from 25 to 10 or 0 °C did not necessarily improve the enantioselectivity of the fluorination (Table 1, entries 16 and 17).

**Table 1:** Asymmetric fluorination of **1a**.^a^

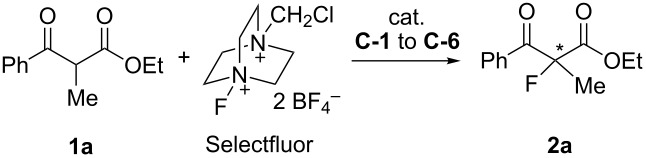					

Entry	Cat. (equiv)	Solvent	*t* (h)	Yield (%)	ee (%)

1	**C-1** (1.0)	MeCN	72	49	65
2	**C-2** (1.0)	MeCN	72	52	56
3	**C-3** (1.0)	MeCN	72	54	51
4	**C-4** (1.0)	MeCN	72	62	46
5	**C-5** (1.0)	MeCN	72	65	48
6	**C-6** (1.0)	MeCN	96	41	18
7	**C-1** (0.5)	MeCN	60	51	26
8	**C-1** (0.2)	MeCN	60	41	<5
9	–	MeCN	96	35	0
10	**C-1** (1.0)	Toluene	72	16	23
11	**C-1** (1.0)	THF	72	32	41
12	**C-1** (1.0)	H_2_O	96	–	–
13	**C-1** (1.0)	MeCN/THF	60	38	45
14	**C-1** (1.0)	MeCN/CF_3_C_6_H_5_	60	43	59
15	**C-1** (1.0)	MeCN/CH_2_Cl_2_	60	51	70
16^b^	**C-1** (1.0)	MeCN/CH_2_Cl_2_	72	46	69
17^c^	**C-1** (1.0)	MeCN/CH_2_Cl_2_	72	39	71

^a^Reaction temperature 25 °C unless otherwise indicated. ^b^Reaction temperature 10 °C. ^c^Reaction temperature 0 °C.

**Scheme 2:**
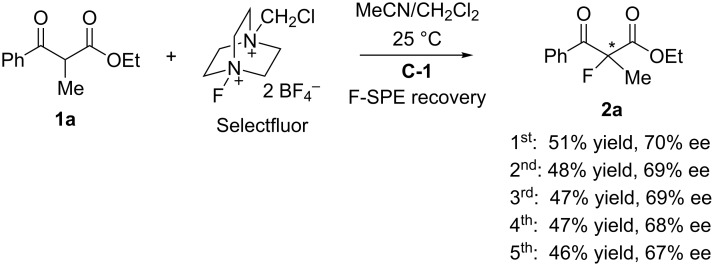
Preparation of **2a** by using recycled quinine ester **C-1**.

Recycling of promoter **C-1** is an important part of this project. In our previous work we have demonstrated that fluorous organocatalysts and reagents can be readily recovered by F-SPE [19–20]. In the current work, upon completion of the fluorination reaction, a base such as aqueous NaOH or KOH was added to the reaction mixture to convert the cinchona alkaloid/Selectfluor complex to free cinchona alkaloid. The organic phase was loaded onto a fluorous silica gel cartridge for F-SPE. Promoter **C-1** was recovered in high yield (94%) and excellent purity (98%). It was used for five rounds without significant change of product yield and ee (Scheme 2).

**Figure 3:**
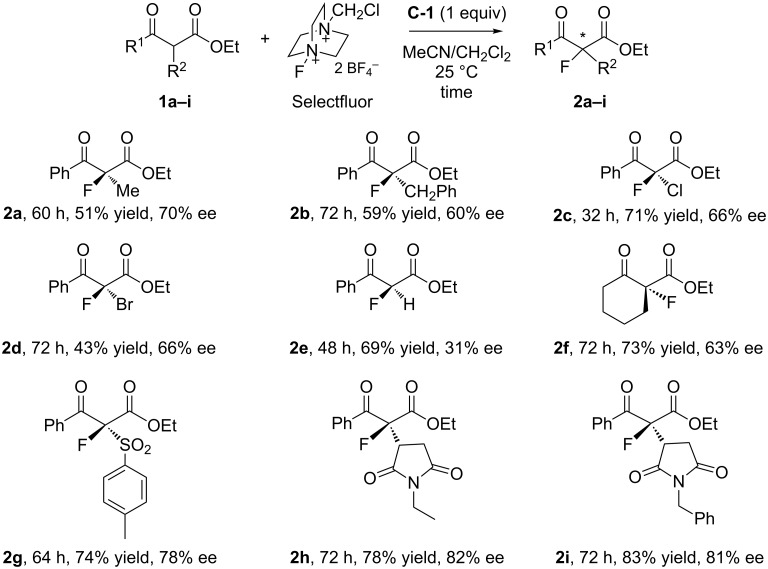
The asymmetric fluorination of various β-ketoesters.

The scope of fluorous quinine ester **C-1-**mediated fluorination was evaluated by carrying out the reactions with a number of α-substituted ethyl benzoylacetates **1a–e** and **1g–i** as well as ethyl 2-cyclohexanonecarboxylate (**1f**). Results summarized in Figure 3 indicate that benzoylacetates bearing R^2^ such as Me, PhCH_2_, Cl, and Br gave fluorination products **2a–d** in 43–71% yields and 60–70% ee. The nonsubstituted benzoylacetate **1e** gave product **2e** in good yield 69% but low ee (31%). Ethyl 2-cyclohexanonecarboxylate (**1f**) afforded product **2f** in 73% yield and 63% ee. Reactions of ethyl benzoylacetates with bigger substitution groups, such as phenylsulfonyl and maleimide derivatives, were also attempted and gave products **2g**–**i** in 74–83% yields and 78–81% ee.

## Conclusion

A fluorous cinchona alkaloid-ester has been introduced as a promoter for Selectfluor-based asymmetric fluorination of β-ketoesters. The fluorous promoter has slightly lower reactivity but better enantioselectivity than the nonfluorous cinchona alkaloids. It can be easily recovered by simple fluorous solid-phase extraction for reuse.

## Experimental

### General

Chemicals and solvents were purchased from commercial suppliers and used as received. ^1^H and ^13^C NMR spectra were recorded on a 300 MHz Varian NMR spectrometer. Chemical shifts were reported in parts per million (ppm), and the residual solvent peak was used as an internal reference, i.e., proton (chloroform δ 7.26), carbon (chloroform δ 77.0). Multiplicity was indicated as follows: s (singlet), d (doublet), t (triplet), q (quartet), m (multiplet), dd (doublet of doublet), br s (broad singlet). Coupling constants were reported in hertz (Hz). LC–MS were performed on an Agilent 2100 system. A C18 column (5.0 μm, 6.0 × 50 mm) was used for the separation. The mobile phases were methanol and water, both containing 0.05% trifluoroacetic acid. A linear gradient was used to increase from 25:75 v/v methanol/water to 100% methanol over 7.0 min at a flow rate of 0.7 mL/min. UV detections were conducted at 210, 254 and 365 nm. Low-resolution mass spectra were recorded in APCI (atmospheric pressure chemical ionization). The high-resolution mass spectra were obtained on a Finnigan/MAT 95XL-T spectrometer. Sorbent silica gel XHL TLC plates (130815) were used for the thin-layer chromatography (TLC). Flash chromatography separations were performed on YAMAZEN AI-580 flash column system with Agela silica gel columns (230–400 μm mesh). The enantiomeric excesses of products were determined by chiral phase HPLC analysis on an SHIMADZU LC-20AD system.

### Synthesis of fluorous quinine ester **C-1**

Thionyl chloride (1.19 g, 10 mmol) was added to a mixture of (1*H*,1*H*,2*H*,2*H*-perfluorooctyl)benzoic acid (0.468 g, 1 mmol) and pyridine (75 mg, 1 mmol). After stirring of the mixture for 4 h at 50 °C, the reaction container was flushed with nitrogen gas to remove unreacted thionyl chloride. Quinine (0.275 g, 0.85 mmol) and *N*,*N*-diisopropylethylamine (129 mg, 1 mmol) in CH_2_Cl_2_ (3 mL) was added, and the solution was stirred for 24 h under reflux. After the reaction had been quenched with H_2_O (2 mL) for 1 h, aqueous K_2_CO_3_ (2 M, 10 mL) was added, and the mixture was extracted with CH_2_Cl_2_ (3 × 10 mL). The CH_2_Cl_2_ layer was washed with aqueous HCl (ca. 2 M, 10 mL) and H_2_O (20 mL). The combined extracts were dried over K_2_CO_3_ and evaporated. The slightly yellow residue was purified by a fluorous silica gel cartridge (5 g). It was first eluted with 80:20 MeOH/H_2_O (20 mL) and then with 100% MeOH. The MeOH fraction was concentrated to give **C-1** as a yellowish solid (0.625 g, 95%). Mp 175–177 °C; ^1^H NMR (CDCl_3_, 300 MHz) δ 1.51–2.05 (m, 6H), 2.30–2.42 (m, 3H), 2.65–2.70 (m, 2H), 2.97–3.18 (m, 4H), 3.50 (q, *J* = 6.9 Hz, 1H), 3.98 (s, 3H), 5.02 (m, 2H), 5.83 (m, 1H), 6.72 (d, *J* = 6.9 Hz, 1H), 7.32–7.51 (m, 5H), 8.01–8.07 (m, 3H), 8.72–8.73 (d, 1H); ^13^C NMR (CDCl_3_, 75 MHz) δ 24.2, 26.5, 27.6, 27.9, 32.4, 39.7, 42.6, 55.6, 56.7, 59.4, 74.5, 101.3, 114.6, 117.3, 118.6, 121.9, 126.9, 128.3, 128.63, 130.2, 131.9, 141.7, 143.6, 144.8, 145.0, 147.5, 156.0, 165.3; APCIMS *m*/*z*: 775.1 (M^+^ + 1); HRMS–ESI (*m*/*z*): [M + H]^+^ calcd. for C_35_H_32_F_13_N_2_O_3_, 775.2205; found, 775.2214.

### Synthesis of quinine benzoate catalyst **C-2**

Benzoyl chloride (28 mg, 0.2 mmol) was added to a mixture of quinine (65 mg, 0.2 mmol) in CH_2_Cl_2_ (0.5 mL). After stirring at rt for 4 h, aqueous K_2_CO_3_ (2 M, 1 mL) was added. The reaction mixture was extracted with CH_2_Cl_2_ (2 × 3 mL). The CH_2_Cl_2_ layer was washed with aqueous HCl (2 M, 2 mL) and H_2_O (3 mL). The combined organic extracts were dried (K_2_CO_3_) and evaporated. The white residue was purified by flash column chromatography (18:1 CH_2_Cl_2_/MeOH) to give quinine benzoate **C-2** (77 mg, 90%) as a colorless solid. ^1^H NMR (CDCl_3_, 300 MHz) δ 1.69–2.00 (m, 5H), 2.42 (m, 1H), 2.82 (m, 2H), 3.19–3.40 (m, 2H), 3.49–3.56 (q, *J* = 7.2 Hz, 1H), 4.00 (s, 3H), 5.04 (m, 2H), 5.82 (m, 1H), 6.97 (d, *J* = 7.2, 1H), 7.40–7.65 (m, 6H), 8.01–8.13 (m, 3H), 8.73 (d, 1H); ^13^C NMR (CDCl_3_, 75 MHz) δ 23.1, 27.2, 27.5, 39.0, 42.5, 56.0, 56.1, 59.0, 73.5, 101.2, 115.2, 117.2, 122.3, 126.6, 127.9, 128.7, 129.6, 129.6, 131.6, 131.8, 133.6, 140.6, 144.7, 147.2, 158.3, 165.1, 200.2; APCIMS *m*/*z*: 429.2 (M^+^ + 1).

### Synthesis of quinine acetate **C-3**

Acetic anhydride (30 mg, 0.3 mmol) was added to a mixture of quinine (65 mg, 0.2 mmol) in CH_2_Cl_2_ (0.5 mL). After stirring at rt for 8 h, aqueous K_2_CO_3_ (2 M, 1 mL) was added, and the mixture was extracted with CH_2_Cl_2_ (2 × 3 mL). The CH_2_Cl_2_ layer was washed with aqueous HCl (2 M, 2 mL) and H_2_O (3 mL). The combined organic extracts were dried (K_2_CO_3_) and evaporated. The white residue was purified by flash column chromatography (18:1 CH_2_Cl_2_/MeOH) to give quinine acetate **C-3** (67 mg, 92%) as a colorless oil. ^1^H NMR (CDCl_3_, 300 MHz) δ 1.26–1.89 (m, 5H), 2.42 (m, 1H), 2.12 (s, 3H), 2.23–2.36 (m, 2H), 2.37–2.70 (m, 2H), 3.00–3.16 (m, 2H), 3.34–3.42 (q, *J* = 7.2 Hz, 1H), 3.96 (s, 3H), 5.03 (m, 2H), 5.86 (m, 1H), 6.50 (d, *J* = 7.2 Hz, 1H), 7.35–7.44 (m, 3H), 8.02 (d, *J* = 9.0 Hz, 3H), 8.74 (d, 1H); ^13^C NMR (CDCl_3_, 75 MHz) δ 21.1, 24.3, 27.5, 27.7, 39.6, 42.4, 55.6, 56.5, 59.0, 73.7, 101.4, 114.5, 118.9, 121.8, 127.0, 131.8, 141.7, 143.5, 144.8, 147.4, 149.6, 157.9, 170.0, 199.5, 200.2; ACPIMS *m*/*z*: 367.2 (M^+^ + 1).

### Synthesis of fluorous pyrrolidine ester **C-6**

*N*,*N*'-Dicyclohexylcarbodiimide (DCC) (0.206 g, 1 mmol) was added to a mixture of (1*H*,1*H*,2*H*,2*H*-perfluorooctyl)benzoic acid (0.468 g, 1 mmol), *N*-Boc-L-prolinol (0.221 g, 1.1 mmol), 4*-*dimethylaminopyridine (DMAP) (0.122 g, 1 mmol) in THF. After being stirred for 24 h at rt, the mixture was directly loaded onto a fluorous silica-gel cartridge (5 g; eluted by 100% methanol) to give the *N*-Boc-L-prolinyl (1*H*,1*H*,2*H*,2*H*-perfluorooctyl)benzoate (0.618 g, 95%). The *N*-Boc ester was then added to a mixture of TFA in CH_2_Cl_2_. After being stirred for 12 h at 0 °C, the reaction mixture was loaded onto a fluorous silica-gel cartridge (5 g) again to give the title compound L-prolinyl (1*H*,1*H*,2*H*,2*H*-perfluorooctyl)benzoate (0.496 g, 90%). ^1^H NMR (CDCl_3_, 300 MHz) δ 1.62–1.89 (m, 3H), 2.16–2.21 (m, 1H), 2.29–2.47 (m, 2H), 2.92–2.98 (m, 2H), 3.48–3.54 (m, 2H), 3.71–3.84 (m, 2H), 4.38–4.42 (m, 1H), 4.95–4.97 (m, 2H), 7.27–7.31 (d, 2H), 7.48–7.50 (d, 2H); ^13^C NMR (CDCl_3_, 75 MHz) δ 25.0, 26.2, 26.3, 26.3, 28.5, 32.2, 32.6, 51.1, 61.6, 67.2, 127.3, 127.6, 128.3, 128.3, 128.4, 130.1, 135.1, 141.4, 171.9; APCIMS *m*/*z*: 552.1 (M^+^ + 1).

### General procedure for fluorination reaction

A mixture of Selectfluor (0.057 g, 0.16 mmol) and fluorous quinine ester **C-1** (0.124 g, 0.16 mmol) in CH_3_CN and CH_2_Cl_2_ was stirred at rt for 1 h. Ethyl 2-methyl-3-oxo-3-phenylpropanoate (**1a**) (0.033 g, 0.16 mmol) was added. After stirring of the mixture at rt for 32 h, the reaction was quenched with H_2_O. After F-SPE, the mixture was extracted with EtOAc. The organic layer was washed with aqueous HCl (2 M, 5 mL) and H_2_O (5 mL), and then dried over Na_2_SO_4_. After evaporation of the solvent, the residue was purified by flash column chromatography (8:1 hexane/EtOAc) to give (*S*)-ethyl 2-methyl-2-fluoro-3-oxo-3-phenylpropanoate (**2a**) as a colorless oil.

#### (*S*)-Ethyl 2-methyl-2-fluoro-3-oxo-3-phenylpropanoate (**2a**)

51% yield, 70% ee. The enantiomeric excess was determined by HPLC on (R,R)-WHELK-O1 with hexane/iPrOH (92:8) as the eluent. Flow rate: 0.6 mL/min, λ = 254 nm; *t*_minor_ = 20.132 min, *t*_major_ = 17.924 min; ^1^H NMR (CDCl_3_, 300 MHz) δ 1.00 (t, *J* = 7.2 Hz, 3H), 1.93 (s, 1H), 4.11 (q, *J* = 7.2 Hz, 2H), 7.33–7.38 (m, 2H), 7.46 (m, 1H), 7.90–7.92 (m, 2H); APCIMS *m*/*z*: 225.2 (M^+^ + 1).

#### (*S*)-Ethyl 2-benzyl-2-fluoro-3-oxo-3-phenylpropanoate (**2b**)

59% yield, 60% ee. The enantiomeric excess was determined by HPLC on Regis Chiral 5 Micron with hexane/iPrOH (90:10) as the eluent. Flow rate: 0.8 mL/min, λ = 254 nm; *t*_minor_ = 8.732 min, *t*_major_ = 10.352 min; ^1^H NMR (CDCl_3_, 300 MHz) δ 0.92 (t, *J =* 7.2 Hz, 3H), 3.48 (d, *J* = 14.1 Hz, 1H), 3.67 (d, *J* = 14.1 Hz, 1H), 4.01 (q, *J =* 7.2 Hz*,* 2H), 7.14–7.23 (m, 5H), 7.26 (m, 2H), 7.36 (m, 1H), 7.91 (d, 2H); APCIMS *m*/*z*: 301.1 (M^+^ + 1).

#### (*R*)-Ethyl 2-chloro-2-fluoro-3-oxo-3-phenylpropanoate (**2c**)

71% yield, 66% ee. The enantiomeric excess was determined by HPLC on Regis Chiral 5 Micron with hexane/iPrOH (90:10) as the eluent. Flow rate: 0.8 mL/min, λ = 254 nm; *t*_minor_ = 12.220 min, *t*_major_ = 14.492 min; ^1^H NMR (CDCl_3_, 300 MHz) δ 1.18 (t, *J* = 7.2 Hz, 3H), 4.32 (q, *J* = 7.2 Hz, 2H), 7.47–7.49 (m, 2H), 7.60 (m, 1H), 8.02–8.05 (m, 2H); APCIMS *m*/*z*: 245.0 (M^+^ + 1).

#### (*R*)-Ethyl 2-bromo-2-fluoro-3-oxo-3-phenylpropanoate (**2d**)

43% yield, 66% ee. The enantiomeric excess was determined by HPLC on Regis Chiral 5 Micron with hexane/iPrOH (90:10) as the eluent. Flow rate: 1.2 mL/min, λ = 254 nm; *t*_minor_ = 5.912 min, *t*_major_ = 7.004 min; ^1^H NMR (CDCl_3_, 300 MHz) δ 1.28 (t, *J* = 7.2 Hz, 3H), 4.38 (q, *J* = 7.2 Hz, 2H), 7.48–7.53 (m, 2H), 7.64 (m, 1H), 8.06–8.10 (m, 2H); APCIMS *m*/*z*: 289.0 (M^+^ + 1).

#### (*S*)-Ethyl 2-fluoro-3-oxo-3-phenylpropanoate (**2e**)

69% yield, 31% ee. The enantiomeric excess was determined by HPLC on (R,R)-WHELK-O1with hexane/iPrOH (95:5) as the eluent. Flow rate: 1.0 mL/min, λ = 254 nm; *t*_minor_ = 5.904 min, *t*_major_ = 5.380 min; ^1^H NMR (CDCl_3_, 300 MHz) δ 1.15 (t, *J* = 7.2 Hz, 3H), 4.23 (q, *J* = 7.2 Hz, 2H), 5.70–5.87 (s, *J* = 48.9 Hz, 1H), 7.18–7.45 (m, 2H), 7.53 (m, 1H), 7.94–7.98 (m, 2H); APCIMS *m*/*z*: 211.1 (M^+^ + 1).

#### (*R*)-Ethyl 2-fluoro-2-cyclohexanonecarboxylate (**2f**)

73% yield, 63% ee. The enantiomeric excess was determined by HPLC on (R,R)-WHELK-O1 with hexane/iPrOH (90:10) as the eluent. Flow rate: 0.8 mL/min, λ = 210 nm; *t*_minor_ = 10.848 min, *t*_major_ = 12.440 min; ^1^H NMR (CDCl_3_, 300 MHz) δ 1.32 (t, *J* = 7.2 Hz, 3H), 1.61–1.89 (m, 2H), 2.06–2.10 (m, 1H), 2.51–2.73 (m, 3H), 4.30 (q, *J* = 7.2 Hz, 2H).

#### (*S*)-Ethyl 2-(4’-methylbenzenesulfonyl)-2-fluoro-3-oxo-3-phenylpropanoate (**2g**)

74% yield, 78% ee. The enantiomeric excess was determined by HPLC on Venusil Chiral OD-H with hexane/iPrOH (92:8) as the eluent. Flow rate: 0.3 mL/min, λ = 254 nm; *t*_minor_ = 27.176 min, *t*_major_ = 24.288 min; ^1^H NMR (CDCl_3_, 300 MHz) δ 1.41 (t, *J =* 7.2 Hz, 3H), 2.36 (s, 3H), 4.38 (q, *J* = 7.2 Hz, 2H), 7.09–7.18 (m, 4H), 7.20–7.35 (m, 3H), 7.54 (s, 2H); APCIMS *m*/*z*: 365.1 (M^+^ + 1).

#### (*S*)-Ethyl 2-(*N*-ethylmaleimide)-2-fluoro-3-oxo-3-phenylpropanoate (**2h**)

78% yield, 82% ee. The enantiomeric excess was determined by HPLC on Venusil Chiral OD-H with hexane/iPrOH (94:6) as the eluent. Flow rate: 0.5 mL/min, λ = 254 nm; *t*_minor_ = 13.832 min, *t*_major_ = 12.432 min; ^1^H NMR (CDCl_3_, 300 MHz) δ 1.16 (t, *J* = 7.2 Hz, 3H), 1.28 (t, *J* = 7.2 Hz, 3H), 2.58 (dd, *J* = 18.3 Hz, 1H), 3.05 (dd, *J* = 18.3 Hz, 1H), 3.61 (q, *J* = 7.2 Hz, 2H), 4.14 (m, 1H ), 4.42 (m, *J* = 7.2 Hz, 2H), 7.46–7.51 (m, 2H), 7.62 (m, 1H), 8.12 (m, 2H); ^13^C NMR (CDCl_3_, 75 MHz) δ 12.9, 13.8, 30.8, 34.1, 44.9, 63.5, 128.5, 128.8, 130.1, 130.2, 134.8, 168.5, 174.8; APCIMS *m*/*z*: 336.1 (M^+^ + 1).

#### (*S*)-Ethyl 2-(*N*-benzylmaleimide)-2-fluoro-3-oxo-3-phenylpropanoate (**2i**)

83% yield, 81% ee. The enantiomeric excess was determined by HPLC on Venusil Chiral OD-H with hexane/iPrOH (92:8) as the eluent. Flow rate: 0.3 mL/min, λ = 254 nm: *t*_minor_ = 27.176 min, *t*_major_ = 24.288 min; ^1^H NMR (CDCl_3_, 300 MHz) δ 1.27 (t, *J* = 7.2 Hz, 3H), 2.58 (dd, *J* = 18.3 Hz, 1H), 3.05 (dd, *J* = 18.3 Hz, 1H), 4.14 (m, 1H ), 4.42 (m, *J* = 7.2 Hz, 2H), 7.67 (q, *J* = 15 Hz, 2H), 7.25–7.37 (m, 5H), 7.46–7.51 (m, 2H), 7.62 (m, 1H), 8.12 (m, 2H); ^13^C NMR (CDCl_3_, 75 MHz) δ 13.8, 30.8, 42.7, 45.0, 45.3, 63.5, 128.0, 128.6, 128.7, 128.8, 130.1, 130.2, 134.9, 174.6; APCIMS *m*/*z*: 398.1 (M^+^ + 1).

### Synthesis of racemic samples

The mixture of Selectfluor (0.057 g, 0.16 mmol) and ethyl benzoylacetate (0.031 g, 0.16 mmol) in CH_3_CN (1 mL) was stirred at 90 °C under microwave irradiation for 40 min. The reaction was quenched by water. The mixture was extracted with ethyl acetate (3 mL). The organic layer was washed with aqueous HCl (2 M, 5 mL) and water (5 mL), and dried over Na_2_SO_4_. After evaporation of the solvent, the residue was purified by flash column chromatography (8:1 hexane/EtOAc) to give ethyl 2-methyl-2-fluoro-3-oxo-3-phenylpropanoate (0.032 g, 94%) as a colorless oil.

### General procedure for recycling of **C-1**

The reaction mixture was loaded onto a fluorous silica-gel cartridge (5 g) and eluted by 80:20 MeOH/H_2_O to collect nonfluorous components, including the fluorinated product. The cartridge was eluted with MeOH to collect **C-1**. After concentration of the MeOH fraction and drying at 60 °C for 8 h, the recovered promoter was ready for the next round of reactions.

## Supporting Information

File 1 Chiral HPLC chromatograms for fluorination products **2a–i**. LC–MS, NMR spectra for fluorination products **2a–i** and cinchona alkaloid derivatives **C-1**, **C-2**, **C-3** and **C-6.** LC–MS spectra for **2h** and HRMS spectra for **C-1**.
