# Supplementary material for: Recyclable fluorous cinchona alkaloid ester as a chiral promoter for asymmetric fluorination of β-ketoesters
Source: Beilstein J Org Chem. 2012 Aug 3;8:1233–40. doi: 10.3762/bjoc.8.138 (PMC3458743; doi:10.3762/bjoc.8.138)

Supporting Information

for

Recyclable fluorous cinchona alkaloid ester as a chiral promoter for asymmetric fluorination of β-ketoesters

Wen-Bin Yi[*1], Xin Huang[2], Zijuan Zhang[2], Dian-Rong Zhu[2], Chun Cai[1], Wei Zhang[*2]

Address: [1]School of Chemical Engineering, Nanjing University of Science and Technology, Xiao Ling Wei Street, Nanjing 210094, People's Republic of China and [2]Department of Chemistry, University of Massachusetts Boston, 100 Morrissey Boulevard, Boston, MA 02125, USA

Email: Wen-Bin Yi - yiwenbin@mail.njust.edu.cn; Wei Zhang - wei2.zhang@umb.edu

*Corresponding author

Chiral HPLC chromatograms for fluorination products 2a–i. LC–MS, NMR spectra for fluorination products 2a–i and cinchona alkaloid derivatives C-1, C-2, C-3 and C-6. LC–MS spectra for 2h and HRMS spectra for C-1.

Table of contents

1. HPLC chromatograms of fluorination products S2

2. HRMS of **C-1**...S11

3. LC-MS spectra of product **2h** S12

4. NMR spectra of catalysts and fluorination products...S13

1. HPLC Chromatogram of Fluorination Products

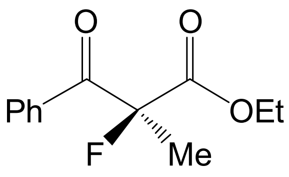

2a

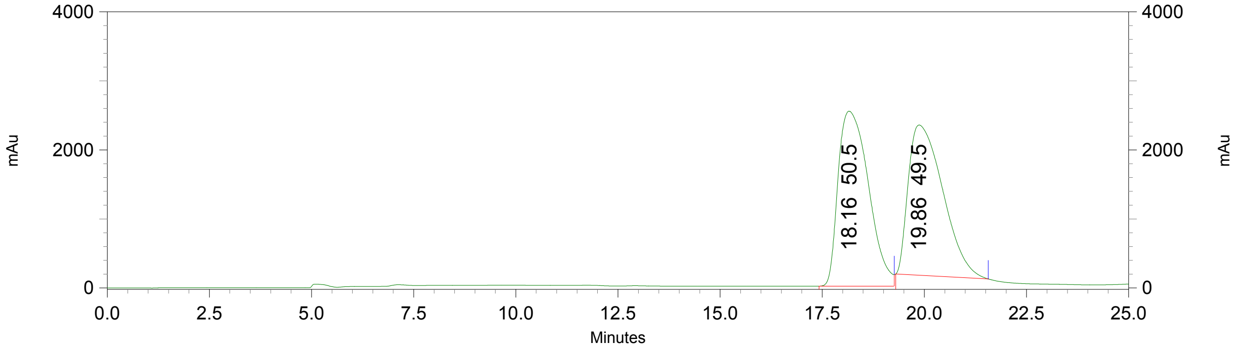

Retention Time	Area	Area %	Height	Height %
18.160	130876531	50.47	2534724	53.81
19.864	128451886	49.53	2176088	46.19
Totals	259328417	100.00	4710812	100.00

Racemic sample

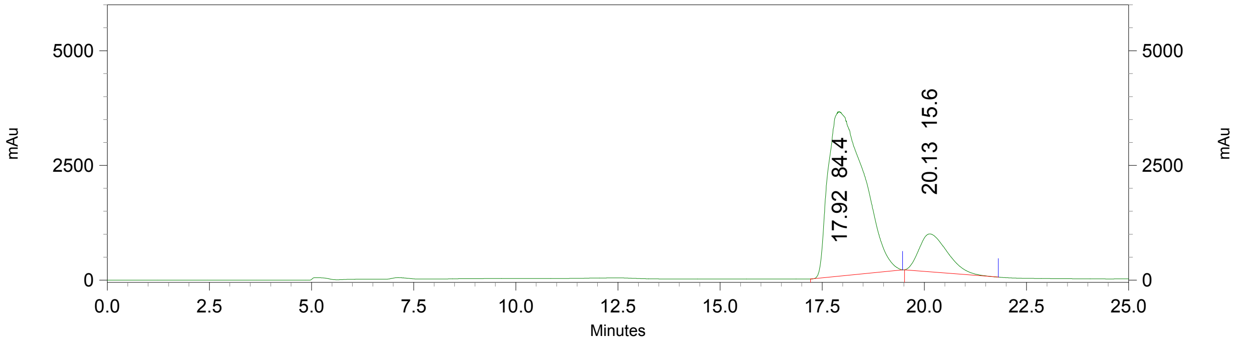

Retention Time	Area	Area %	Height	Height %
17.924	217622833	84.43	3586151	81.27
20.132	40123778	15.57	826271	18.73
Totals	257746611	100.00	4412422	100.00

Chiral sample

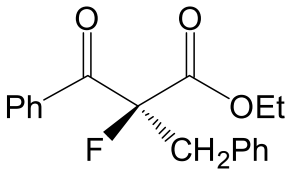

2b

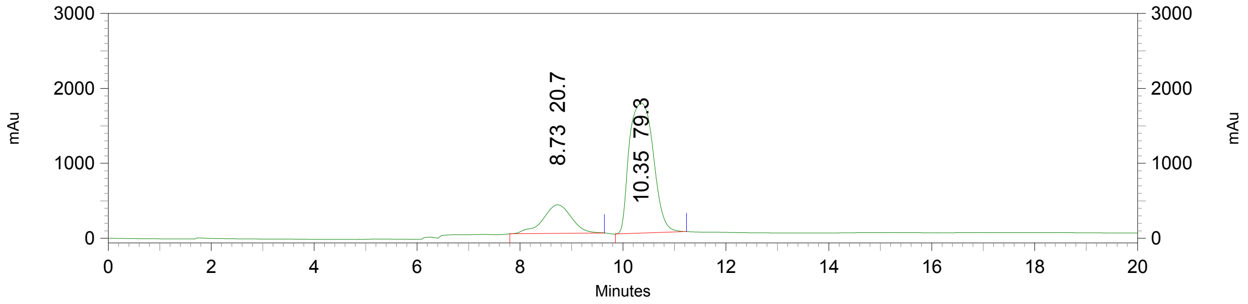

Retention Time	Area	Area %	Height	Height %
8.732	14775829	20.68	381327	18.03
10.352	56661484	79.32	1733272	81.97
Totals	71437313	100.00	2114599	100.00

Chiral sample

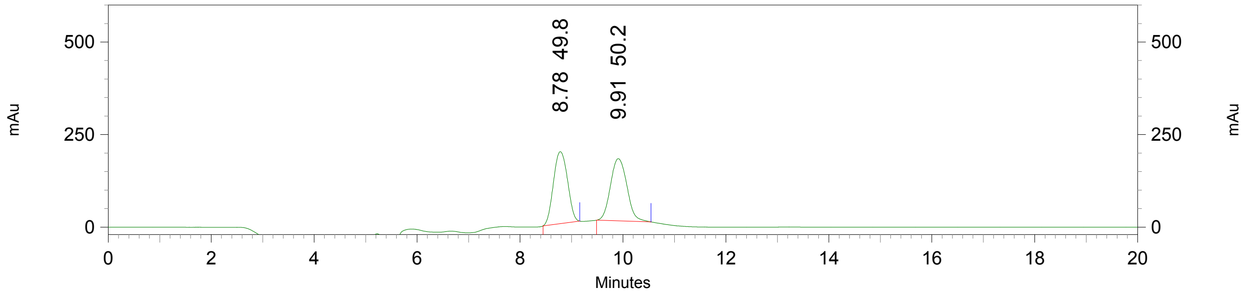

Retention Time	Area	Area %	Height	Height %
8.784	3699041	49.75	194445	53.63
9.912	3735561	50.25	168094	46.37
Totals	7434602	100.00	362539	100.00

Racemic sample

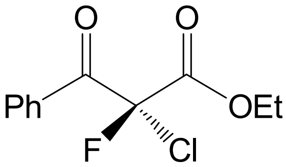

2c

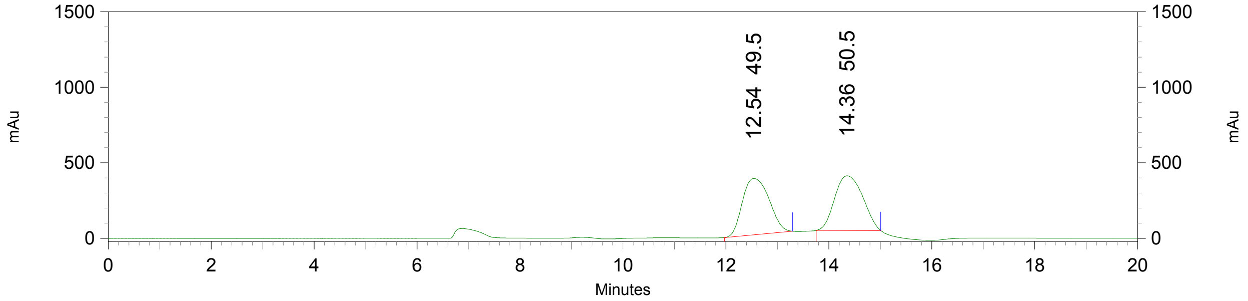

Retention Time	Area	Area %	Height	Height %
12.544	13445373	49.54	373360	50.78
14.356	13692870	50.46	361825	49.22
Totals	27138243	100.00	735185	100.00

Racemic sample

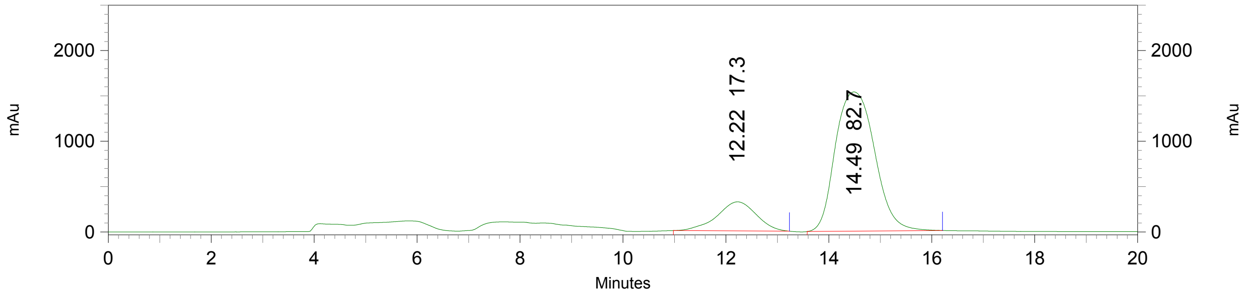

Retention Time	Area	Area %	Height	Height %
12.220	17007049	17.27	320425	17.28
14.492	81483764	82.73	1534387	82.72
Totals	98490813	100.00	1854812	100.00

Chiral sample

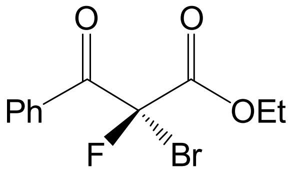

2d

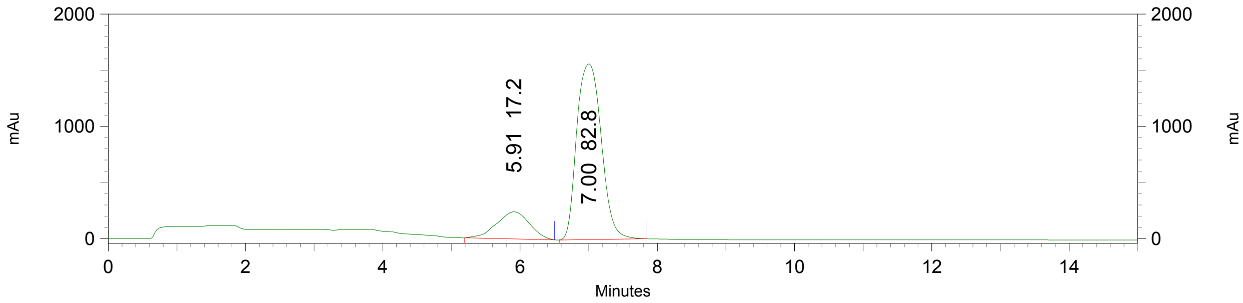

Retention Time	Area	Area %	Height	Height %
5.912	8053638	17.15	241788	13.39
7.004	38894427	82.85	1563960	86.61
Totals	46948065	100.00	1805748	100.00

Chiral sample

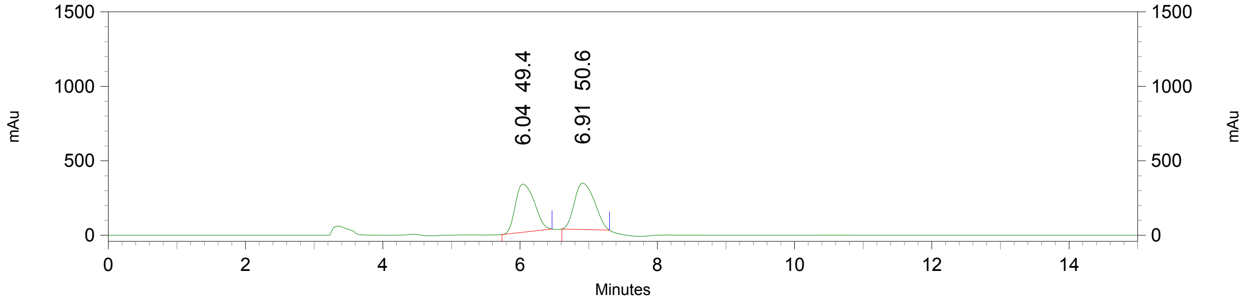

Retention Time	Area	Area %	Height	Height %
6.044	6190037	49.40	323087	50.94
6.912	6341011	50.60	311149	49.06
Totals	12531048	100.00	634236	100.00

Racemic sample

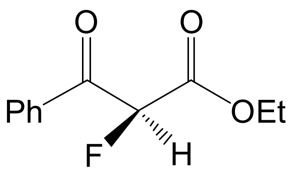

2e

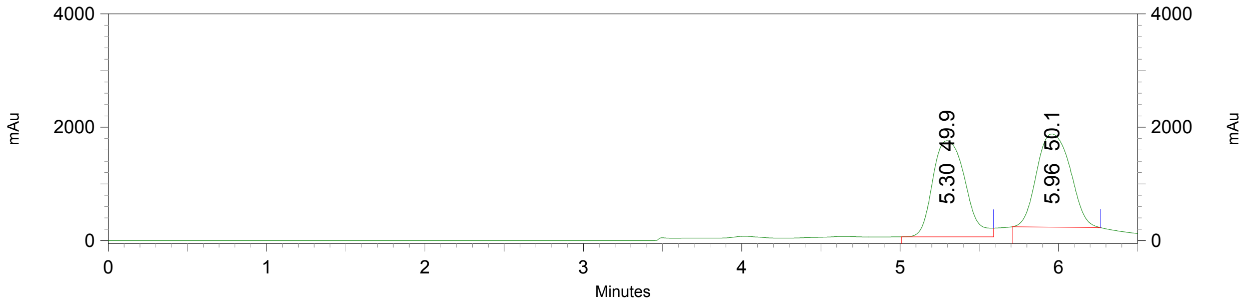

Retention Time	Area	Area %	Height	Height %
5.296	24226523	49.93	1693699	50.77
5.960	24296012	50.07	1642511	49.23
Totals	48522535	100.00	3336210	100.00

Racemic sample

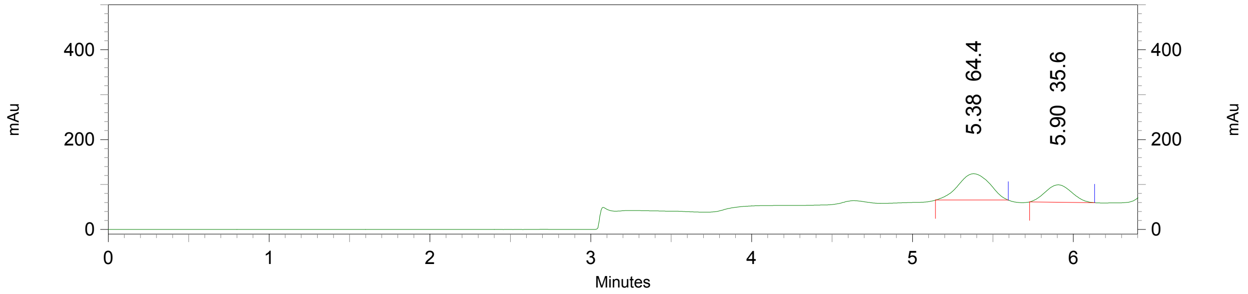

Retention Time	Area	Area %	Height	Height %
5.380	797907	64.43	58574	60.19
5.904	440552	35.57	38740	39.81
Totals	1238459	100.00	97314	100.00

Chiral sample

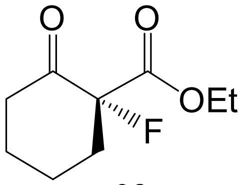

2f

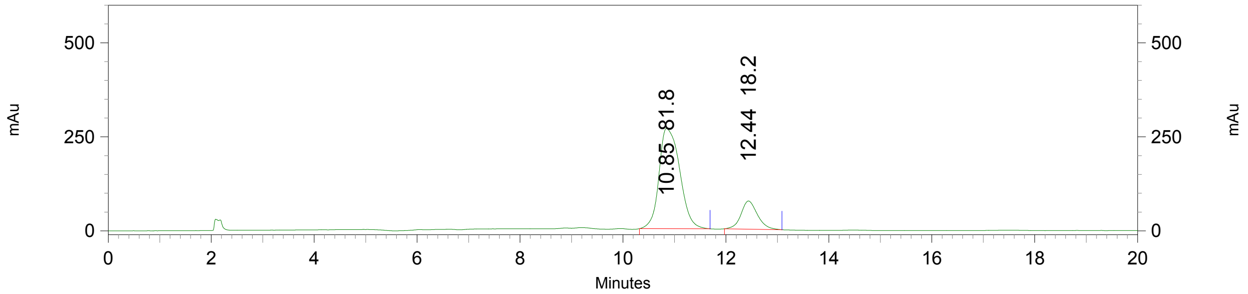

Retention Time	Area	Area %	Height	Height %
10.848	7476587	81.80	267008	78.07
12.440	1662976	18.20	75023	21.93
Totals	9139563	100.00	342031	100.00

Chiral sample

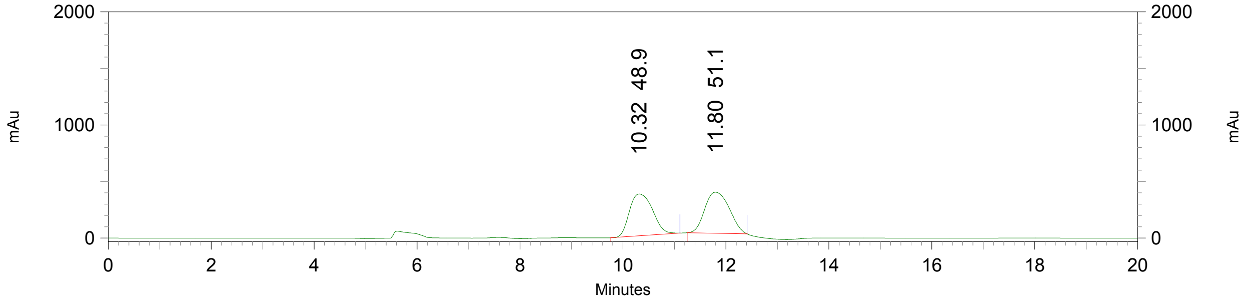

Retention Time	Area	Area %	Height	Height %
10.316	11532128	48.91	369589	50.43
11.800	12044639	51.09	363256	49.57
Totals	23576767	100.00	732845	100.00

Racemic sample

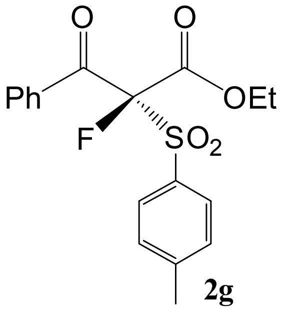

2g

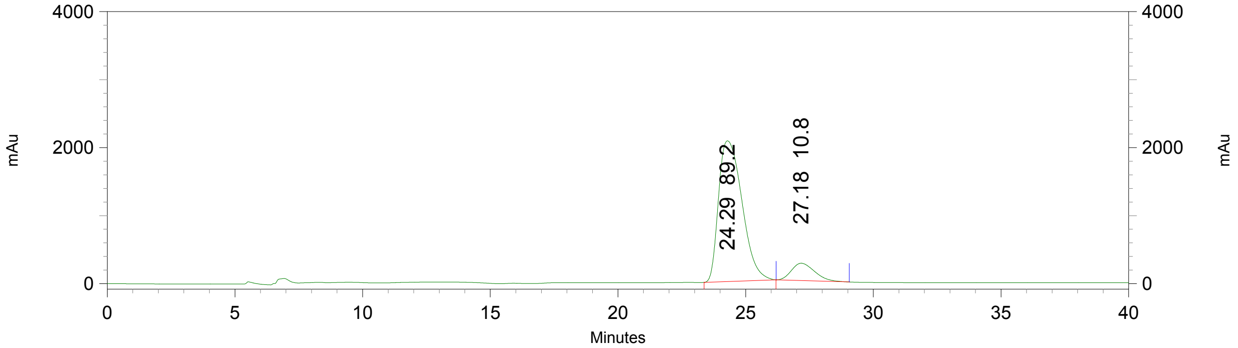

Retention Time	Area	Area %	Height	Height %
24.288	132223497	89.19	2069101	89.03
27.176	16028095	10.81	254896	10.97
Totals	148251592	100.00	2323997	100.00

Chiral sample

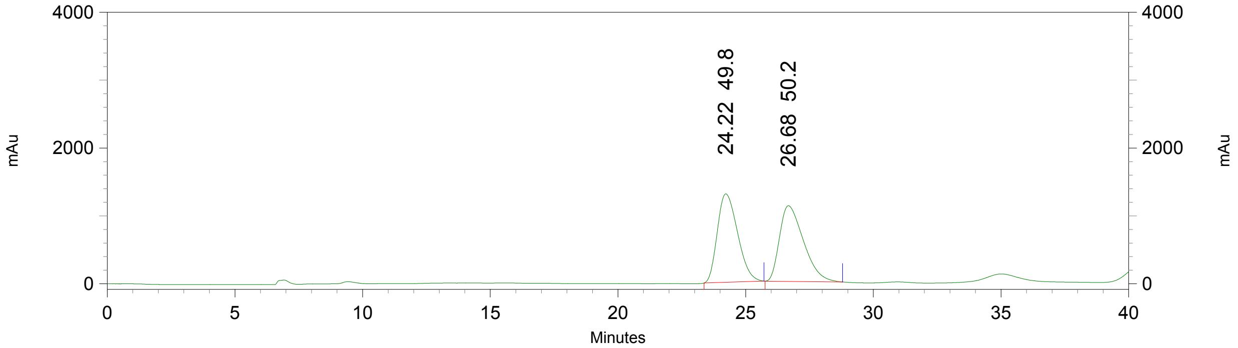

Retention Time	Area	Area %	Height	Height %
24.224	71970053	49.76	1302776	53.90
26.676	72665895	50.24	1114459	46.10
Totals	144635948	100.00	2417235	100.00

Racemic sample

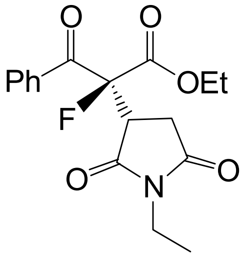

2h

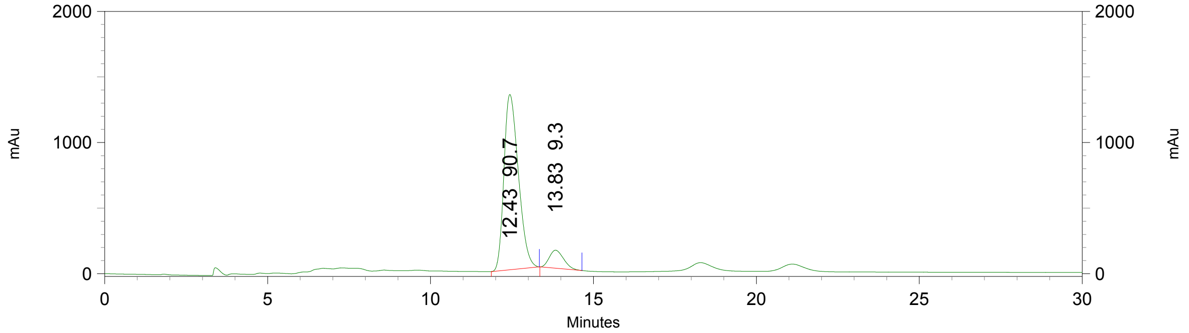

Retention Time	Area	Area %	Height	Height %
12.432	41124380	90.70	1336520	90.64
13.832	4214965	9.30	138013	9.36
Totals	45339345	100.00	1474533	100.00

Chiral sample

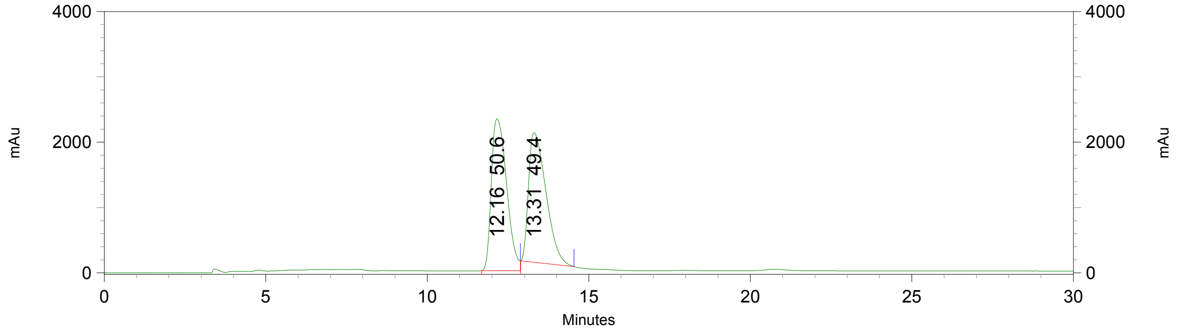

Retention Time	Area	Area %	Height	Height %
12.160	76429475	50.56	2321847	53.94
13.308	74742615	49.44	1982343	46.06
Totals	151172090	100.00	4304190	100.00

Racemic sample

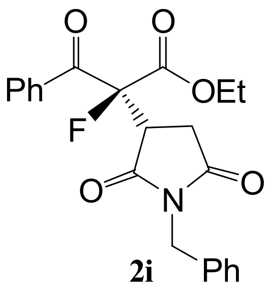

2i

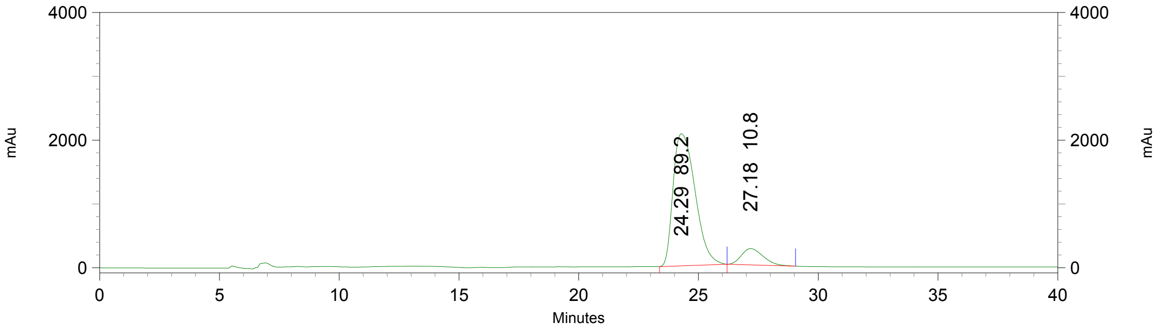

Retention Time	Area	Area %	Height	Height %
24.288	132223497	89.19	2069101	89.03
27.176	16028095	10.81	254896	10.97
Totals	148251592	100.00	2323997	100.00

Chiral sample

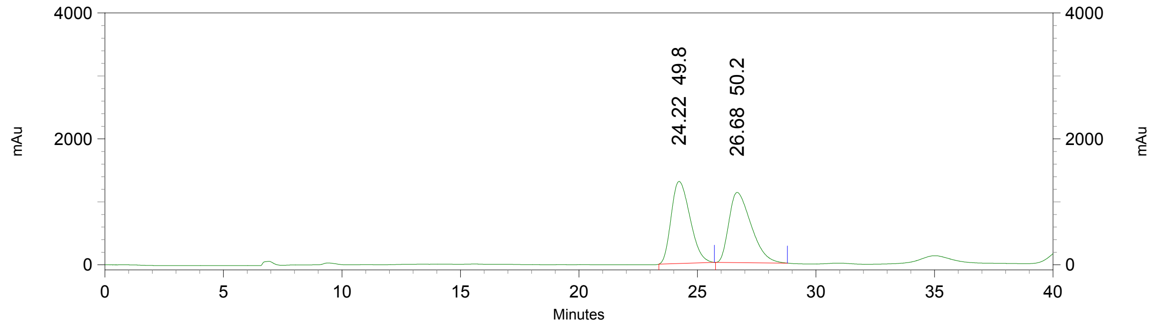

Retention Time	Area	Area %	Height	Height %
24.224	71970053	49.76	1302776	53.90
26.676	72665895	50.24	1114459	46.10
Totals	144635948	100.00	2417235	100.00

Racemic sample

2. HRMS of C-1

Elemental Composition Report **Page 1**

Single Mass Analysis
Tolerance = 10.0 PPM / DBE: min = -1.5, max = 150.0
Element prediction: Off
Number of isotope peaks used for i-FIT = 3

Monoisotopic Mass, Even Electron Ions
300 formula(e) evaluated with 4 results within limits (all results (up to 1000) for each mass)
Elements Used:
C: 0-150 H: 0-200 N: 1-5 O: 1-5 F: 12-13

Kadam, Asha , (2)F-QN University of Illnois, SCS, Mass Spectrometry Lab Q-tof UE521
Qtof_38759 46 (3.293) AM (Cen,3, 80.00, Ar,15000.0,716.46,0.70,LS 3); Sm (SG, 2x3.00); Cm (46:48) 1: TOF MS ES+
9.52e+002

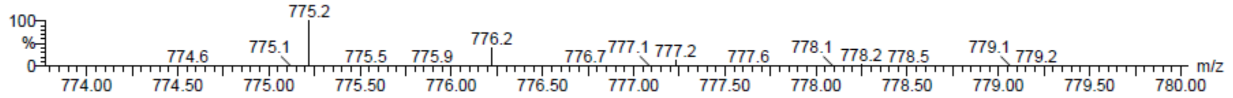

Minimum:				-1.5		
Maximum:		5.0	10.0	150.0		

Mass	Calc. Mass	mDa	PPM	DBE	i-FIT	Formula
775.2214	775.2205	0.9	1.2	14.5	7.5	C35 H32 N2 O3 F13
	775.2194	2.0	2.6	18.5	6.4	C38 H31 N2 O2 F12
	775.2165	4.9	6.3	10.5	12.9	C30 H32 N4 O5 F13
	775.2154	6.0	7.7	14.5	8.5	C33 H31 N4 O4 F12

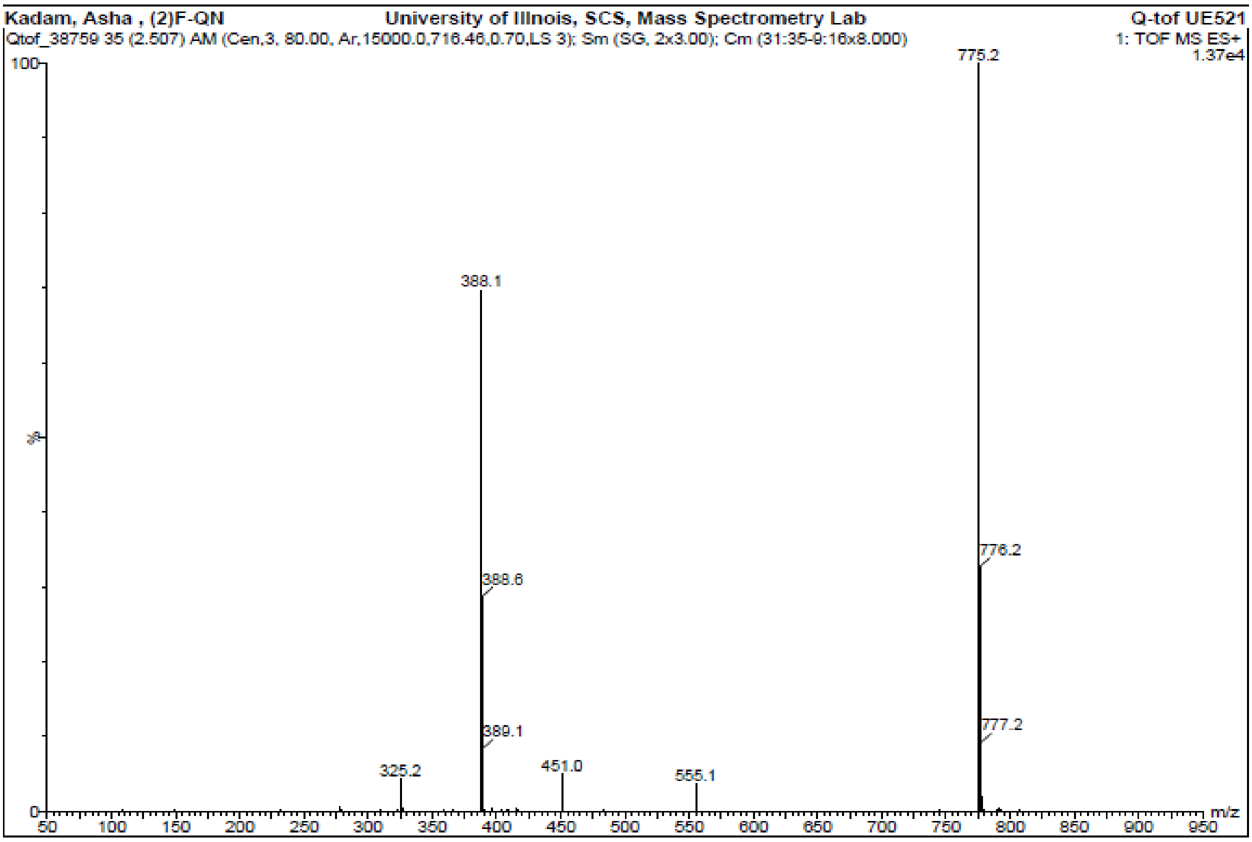

3. LC-MS spectra of product 2h

```
Print of all graphic windows
Data File  : C:\CHEM32\1\DATA\12-04-12\024-0401.D
Sample Name : ethylmalonide
=====================================================================
Acq. Operator   : Asha                          Seq. Line :   4
Acq. Instrument : Instrument 1                   Location : Vial 24
Injection Date  : 4/12/2012 6:27:45 PM                Inj :   1
                                               Inj Volume : 4 µl
Method          : C:\CHEM32\1\METHODS\CINCHONA.M
Last changed    : 3/23/2012 1:40:38 PM by Asha
```

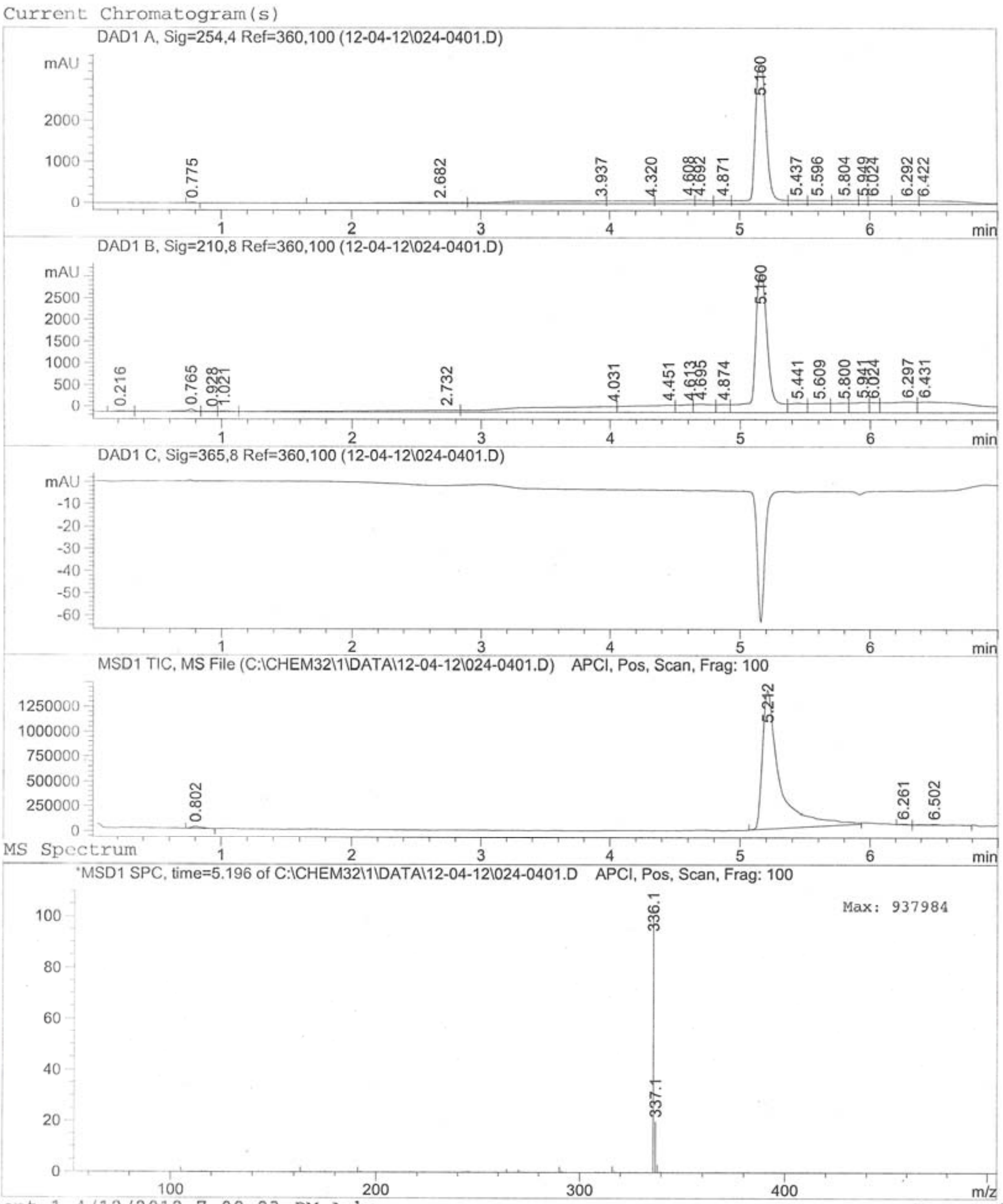

Instrument 1 4/12/2012 7:08:23 PM Asha Page 1 of 1

4. NMR spectra of catalysts and products

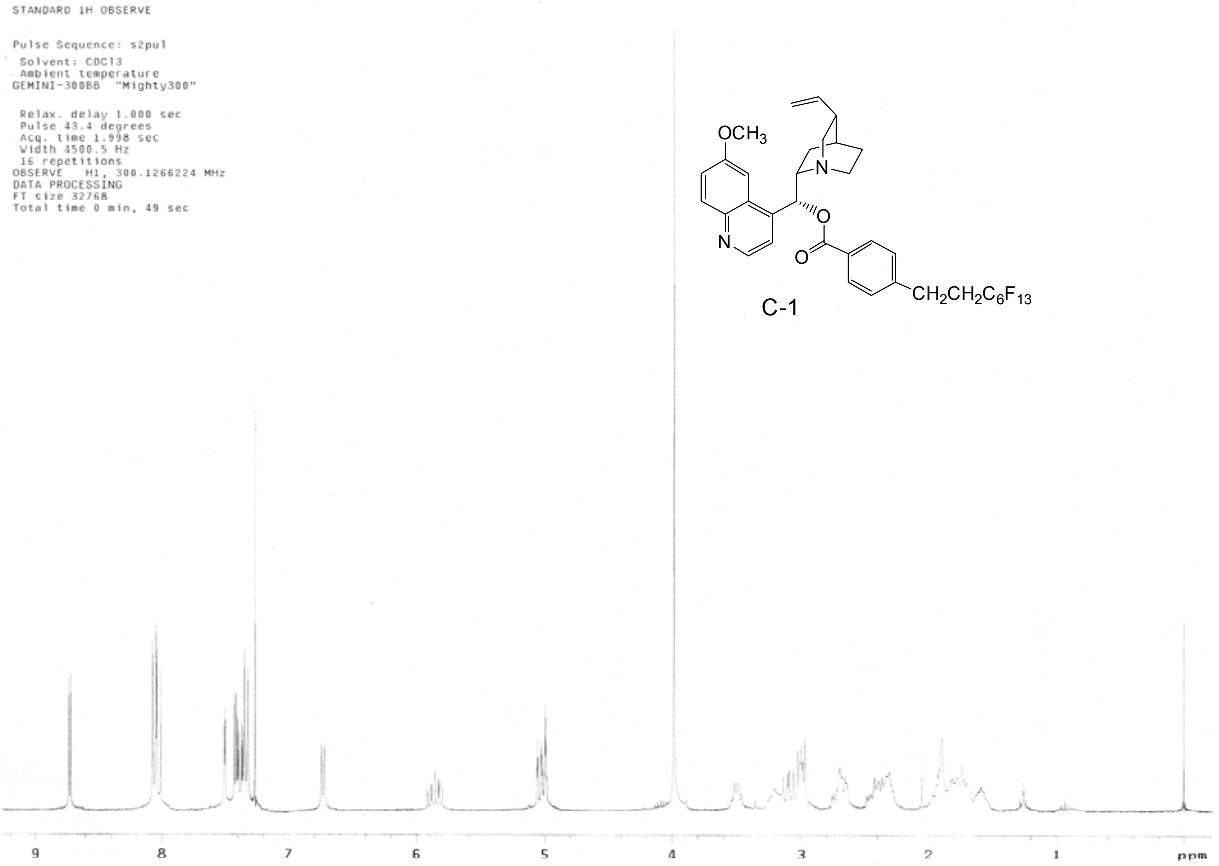

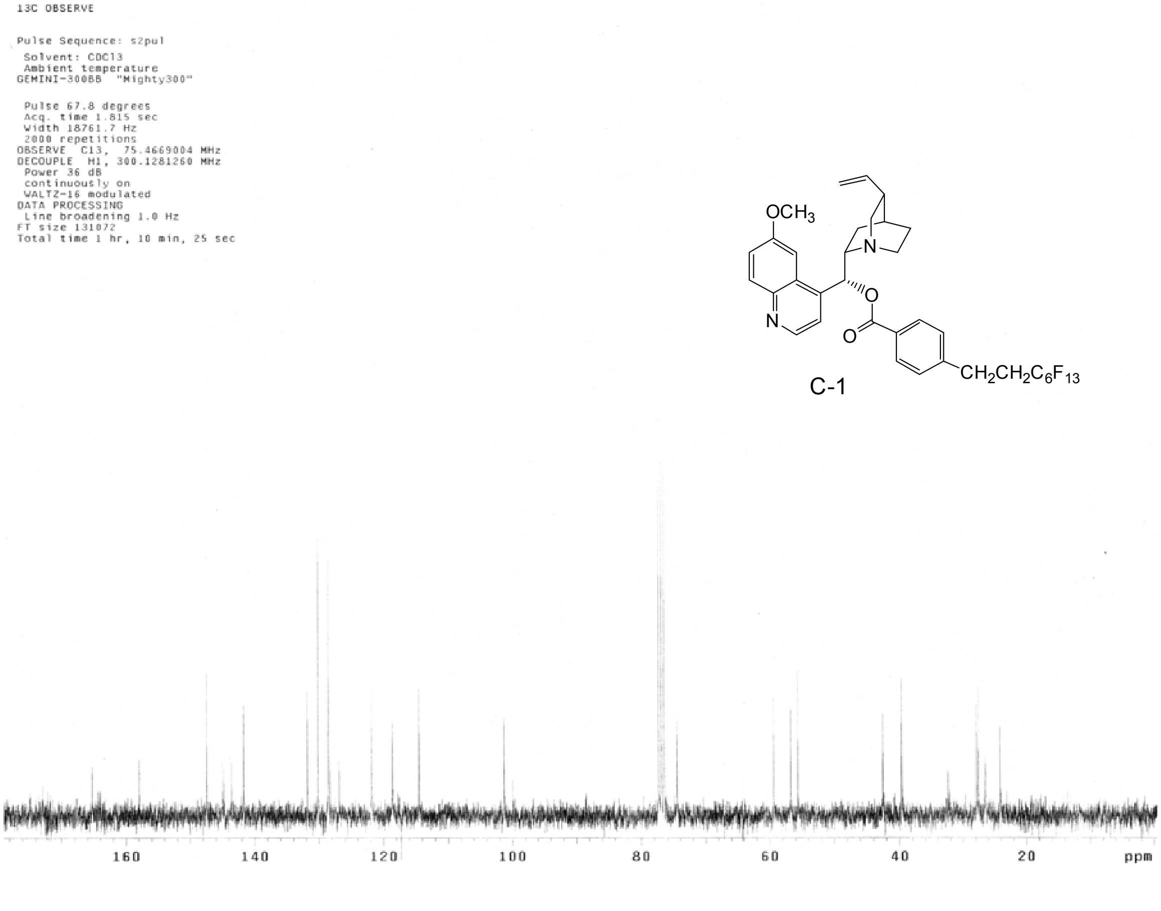

STANDARD 1H OBSERVE

Pulse Sequence: s2pul
Solvent: CDCl3
Ambient temperature
GEMINI-300BB "Mighty300"

Relax. delay 1.000 sec
Pulse 43.4 degrees
Acq. time 1.998 sec
Width 4500.5 Hz
16 repetitions
OBSERVE H1, 300.1266210 MHz
DATA PROCESSING
FT size 32768
Total time 0 min, 49 sec

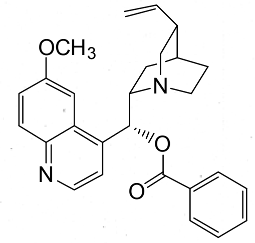

C-2

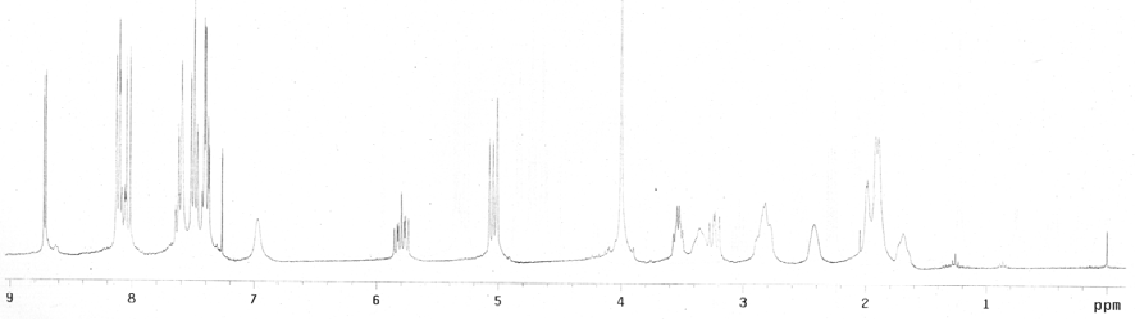

13C OBSERVE

Pulse Sequence: s2pul
Solvent: CDCl3
Ambient temperature
GEMINI-300BB "Mighty300"

Pulse 67.8 degrees
Acq. time 1.815 sec
Width 18761.7 Hz
1024 repetitions
OBSERVE C13, 75.4669021 MHz
DECOUPLE H1, 300.1281260 MHz
Power 36 dB
continuously on
WALTZ-16 modulated
DATA PROCESSING
Line broadening 1.0 Hz
FT size 131072
Total time 36 min, 3 sec

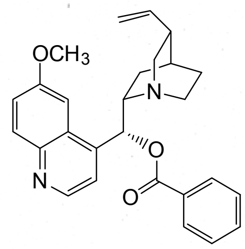

C-2

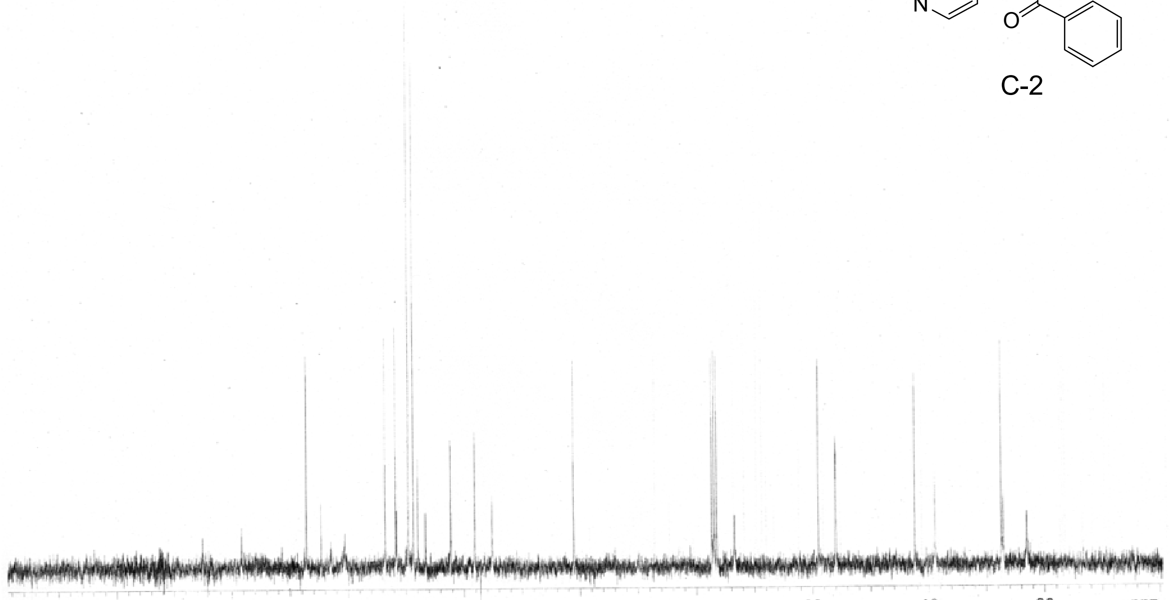

STANDARD 1H OBSERVE

Pulse Sequence: s2pul
Solvent: CDCl3
Ambient temperature
File: QN-acetate
GEMINI-300BB "Mighty300"

Relax. delay 1.000 sec
Pulse 43.4 degrees
Acq. time 1.998 sec
Width 4500.5 Hz
16 repetitions
OBSERVE H1, 300.1266205 MHz
DATA PROCESSING
FT size 32768
Total time 0 min, 49 sec .

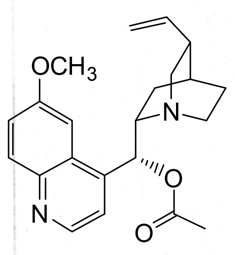

C-3

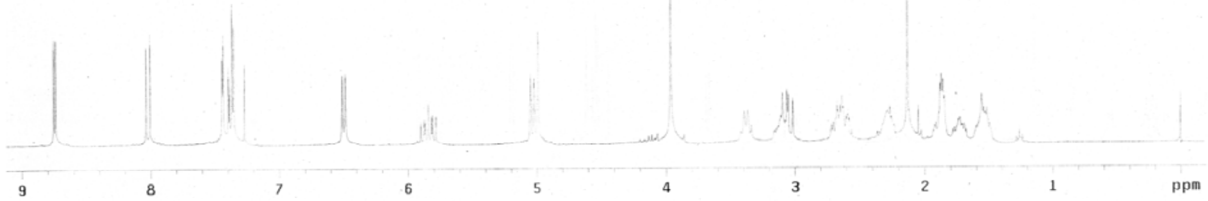

13C OBSERVE

Pulse Sequence: s2pul
Solvent: CDCl3
Ambient temperature
GEMINI-300BB "Mighty300"

Pulse 67.8 degrees
Acq. time 1.815 sec
Width 18761.7 Hz
1024 repetitions
OBSERVE C13, 75.4669015 MHz
DECOUPLE H1, 300.1281260 MHz
Power 36 dB
continuously on
WALTZ-16 modulated
DATA PROCESSING
Line broadening 1.0 Hz
FT size 131072
Total time 36 min, 3 sec

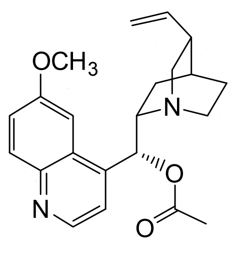

C-3

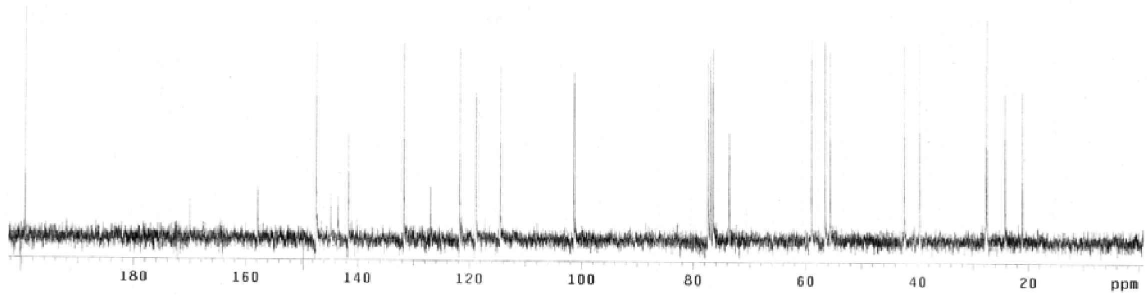

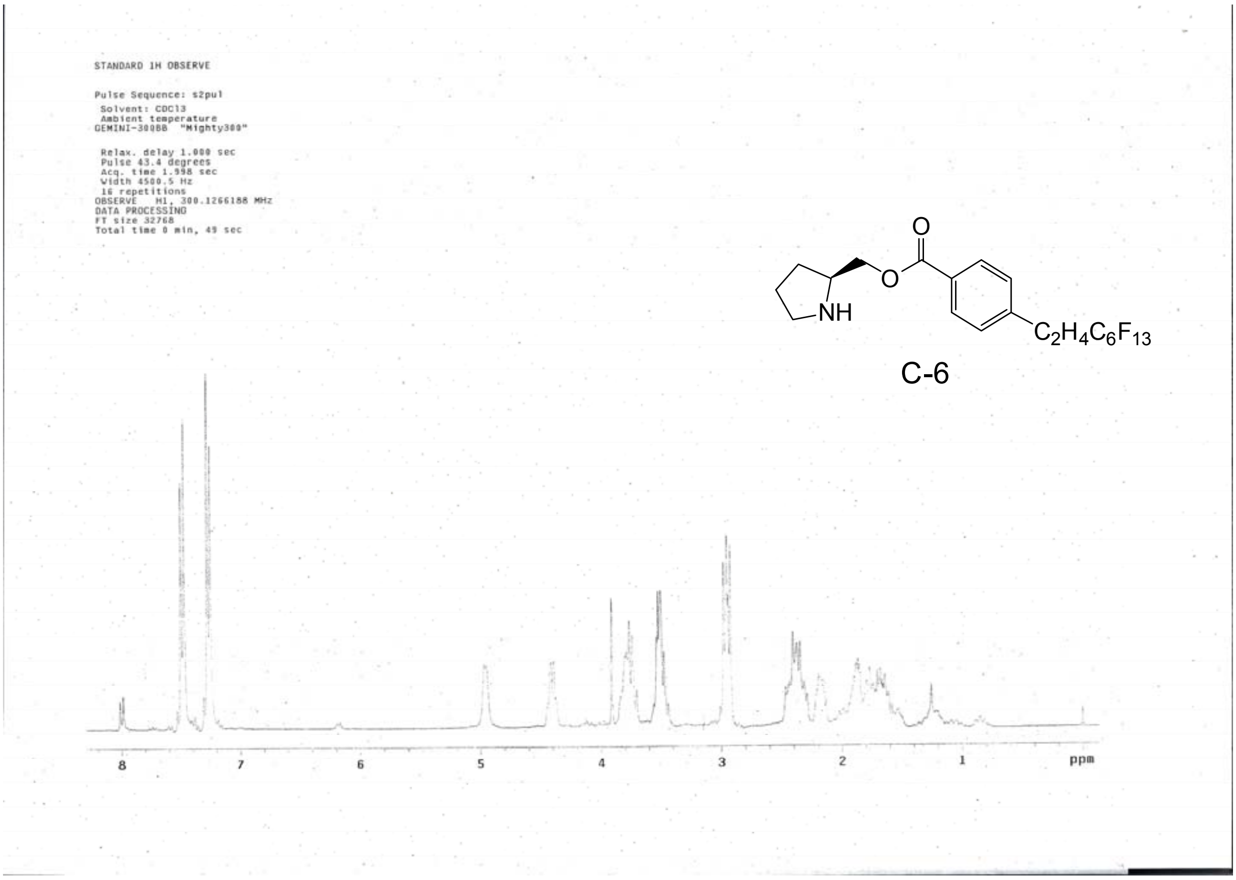
STANDARD 1H OBSERVE
Pulse Sequence: s2pul
Solvent: CDCl3
Ambient temperature
GEMINI-300BB "Mighty300"
Relax. delay 1.000 sec
Pulse 43.4 degrees
Acq. time 1.998 sec
Width 4500.5 Hz
16 repetitions
OBSERVE H1, 300.1266188 MHz
DATA PROCESSING
FT size 32768
Total time 0 min, 49 sec
O
O
NH
$C_2H_4C_6F_{13}$
C-6
8
7
6
5
4
3
2
1
ppm

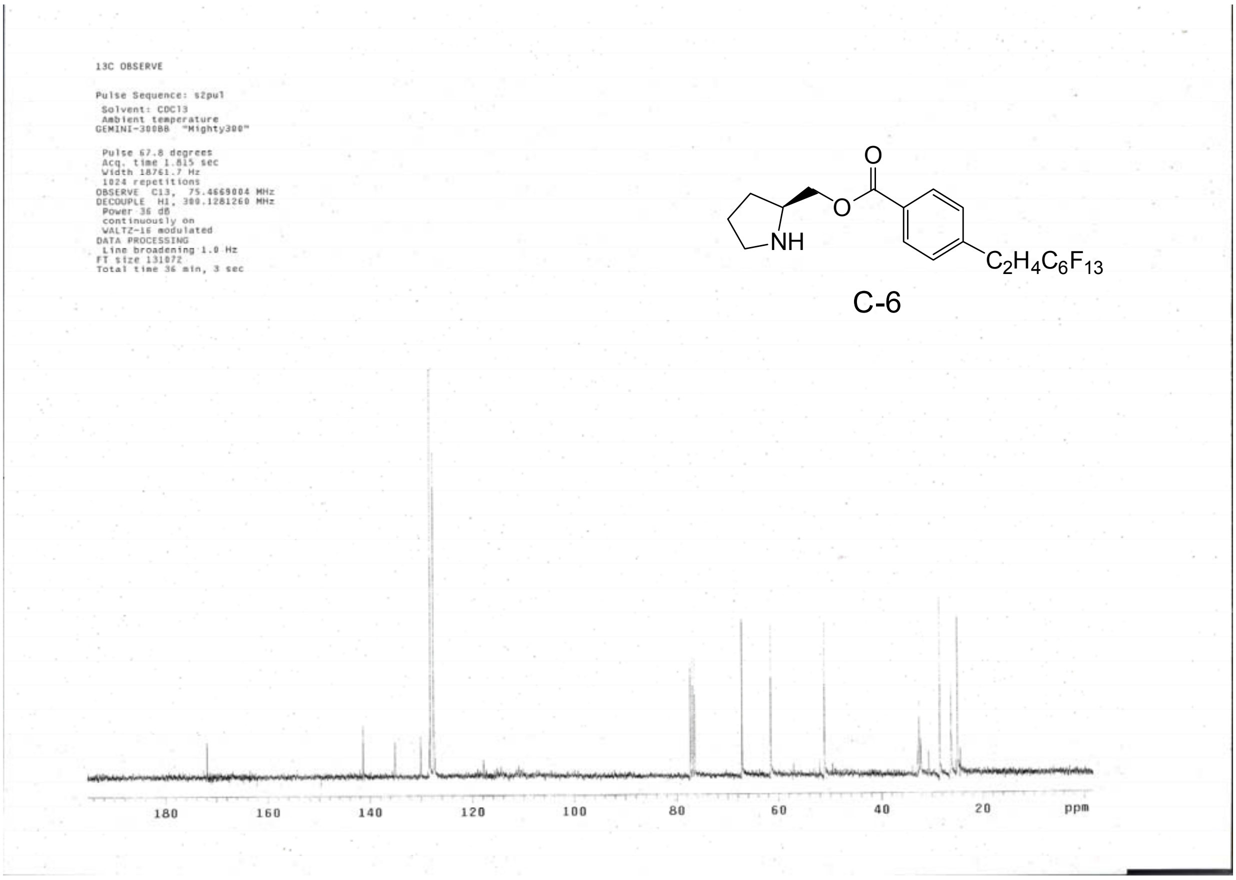
13C OBSERVE
Pulse Sequence: s2pul
Solvent: CDCl3
Ambient temperature
GEMINI-300BB "Mighty300"
Pulse 67.8 degrees
Acq. time 1.815 sec
Width 18761.7 Hz
1024 repetitions
OBSERVE C13, 75.4669004 MHz
DECOUPLE H1, 300.1281260 MHz
Power 36 dB
continuously on
WALTZ-16 modulated
DATA PROCESSING
Line broadening 1.0 Hz
FT size 131072
Total time 36 min, 3 sec
O
O
NH
$C_2H_4C_6F_{13}$
C-6
180
160
140
120
100
80
60
40
20
ppm

STANDARD 1H OBSERVE

Pulse Sequence: s2pul
Solvent: CDCl3
Ambient temperature
GEMINI-300BB "Mighty300"

Relax. delay 1.000 sec
Pulse 43.4 degrees
Acq. time 1.998 sec
Width 4500.5 Hz
16 repetitions
OBSERVE H1, 300.1266478 MHz
DATA PROCESSING
FT size 32768
Total time 0 min, 49 sec

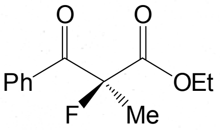

2a

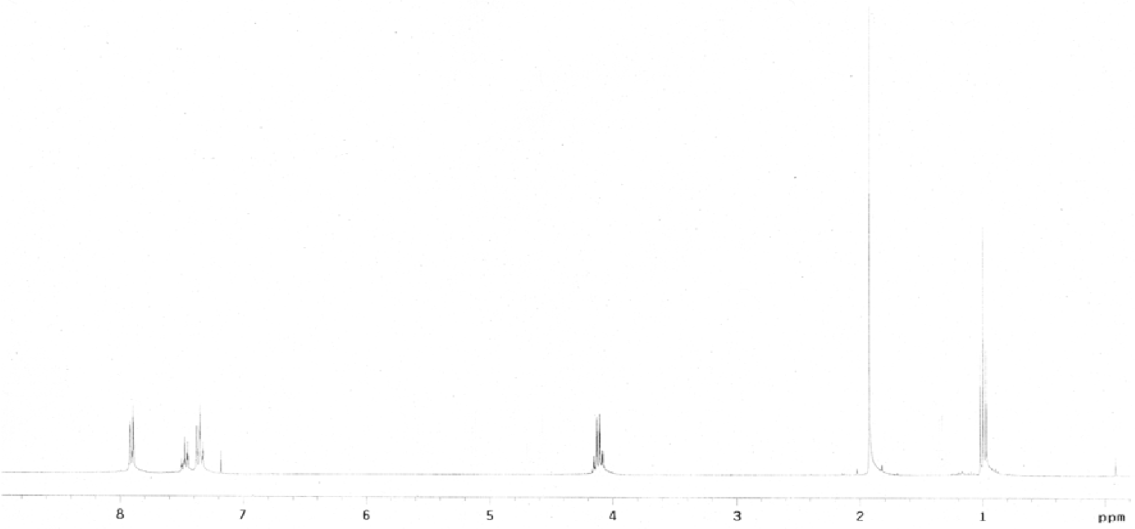

STANDARD 1H OBSERVE

Pulse Sequence: s2pul
Solvent: CDCl3
Ambient temperature
GEMINI-300BB "Mighty300"

Relax. delay 1.000 sec
Pulse 43.4 degrees
Acq. time 1.998 sec
Width 4500.5 Hz
16 repetitions
OBSERVE H1, 300.1266478 MHz
DATA PROCESSING
FT size 32768
Total time 0 min, 0 sec

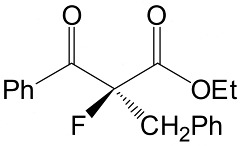

2b

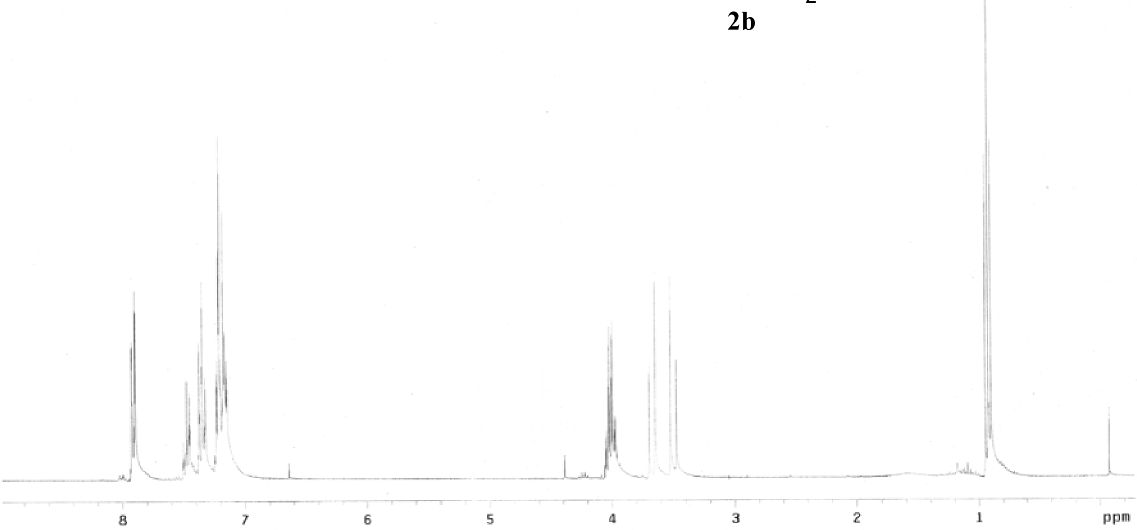

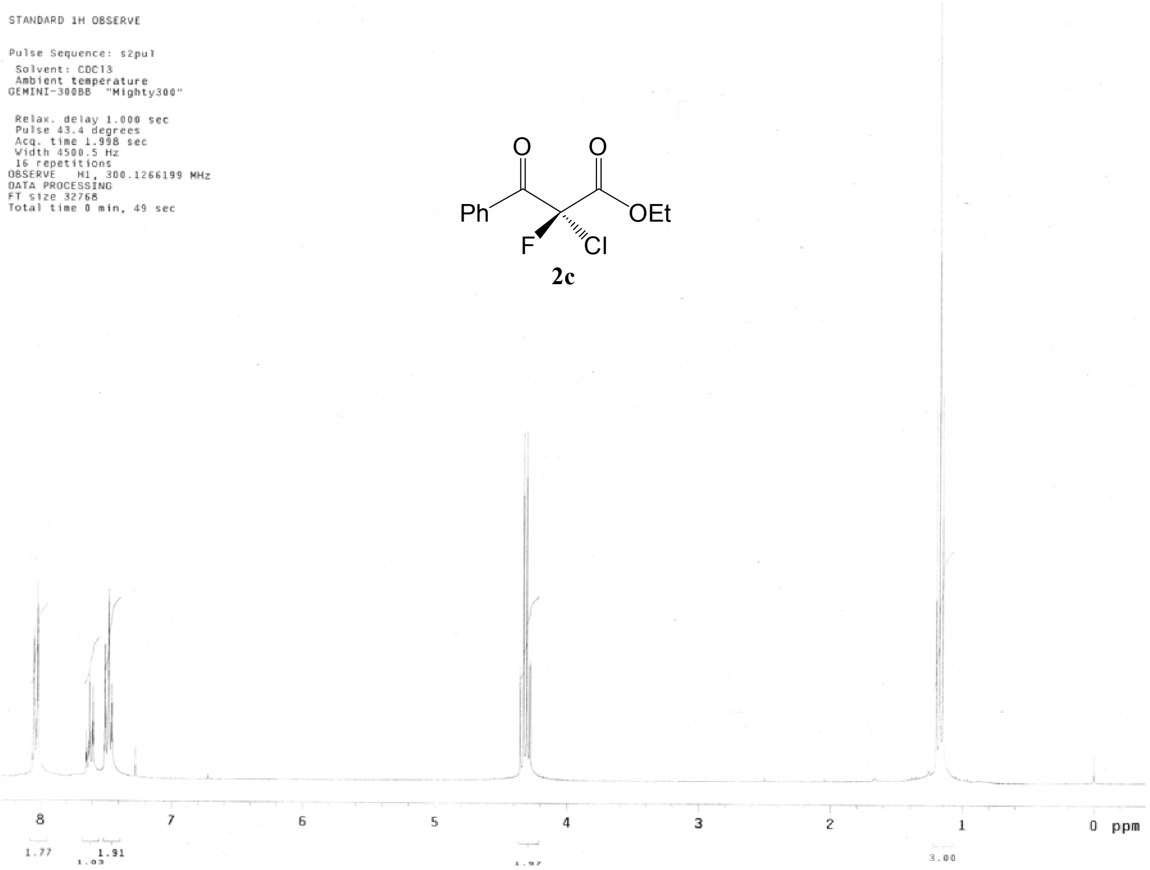
STANDARD 1H OBSERVE
Pulse Sequence: s2pul
Solvent: CDCl3
Ambient temperature
GEMINI-300BB "Mighty300"
Relax. delay 1.000 sec
Pulse 43.4 degrees
Acq. time 1.998 sec
Width 4500.5 Hz
16 repetitions
OBSERVE H1, 300.1266199 MHz
DATA PROCESSING
FT size 32768
Total time 0 min, 49 sec
Ph
O
O
OEt
F
Cl
2c
8 7 6 5 4 3 2 1 0 ppm
1.77
1.91
3.00

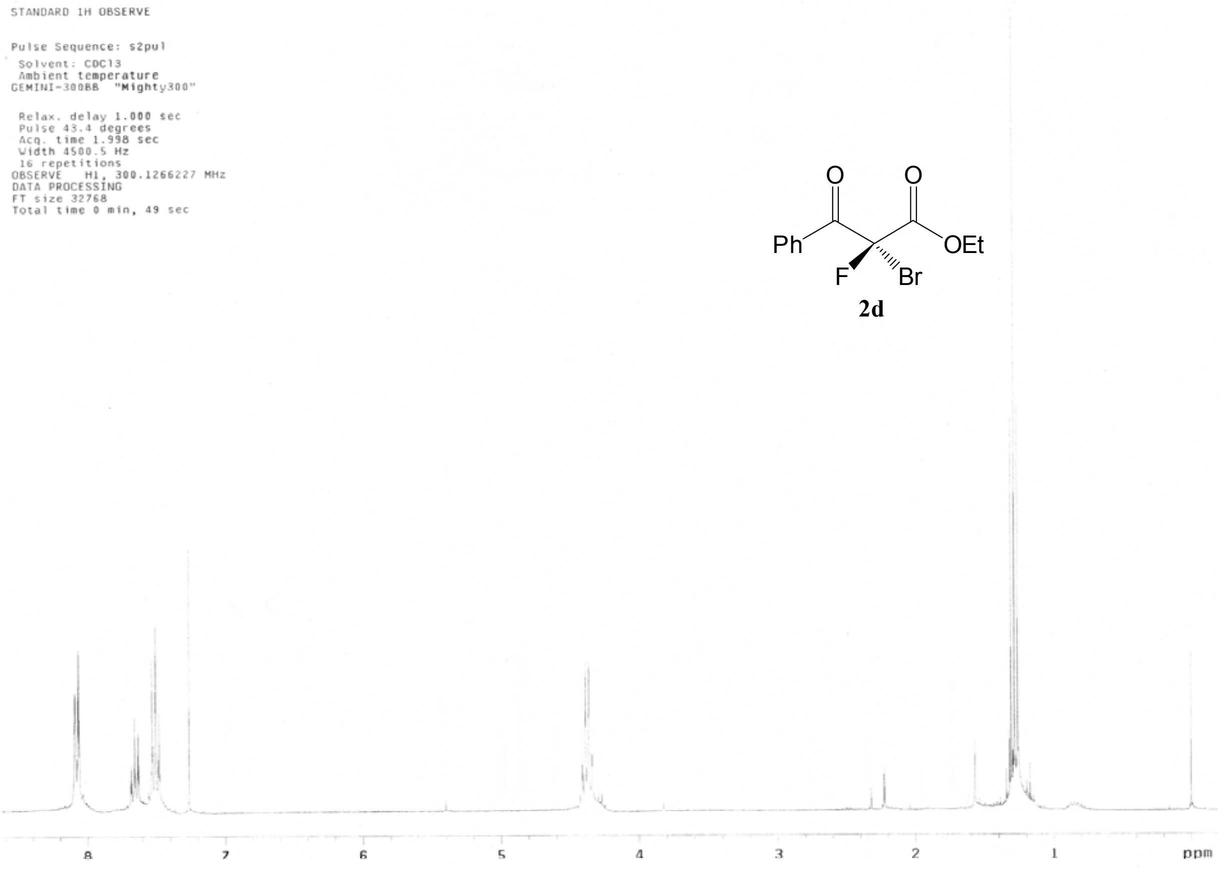
STANDARD 1H OBSERVE
Pulse Sequence: s2pul
Solvent: CDCl3
Ambient temperature
GEMINI-300BB "Mighty300"
Relax. delay 1.000 sec
Pulse 43.4 degrees
Acq. time 1.998 sec
Width 4500.5 Hz
16 repetitions
OBSERVE H1, 300.1266227 MHz
DATA PROCESSING
FT size 32768
Total time 0 min, 49 sec
Ph
O
O
OEt
F
Br
2d
8 7 6 5 4 3 2 1 ppm

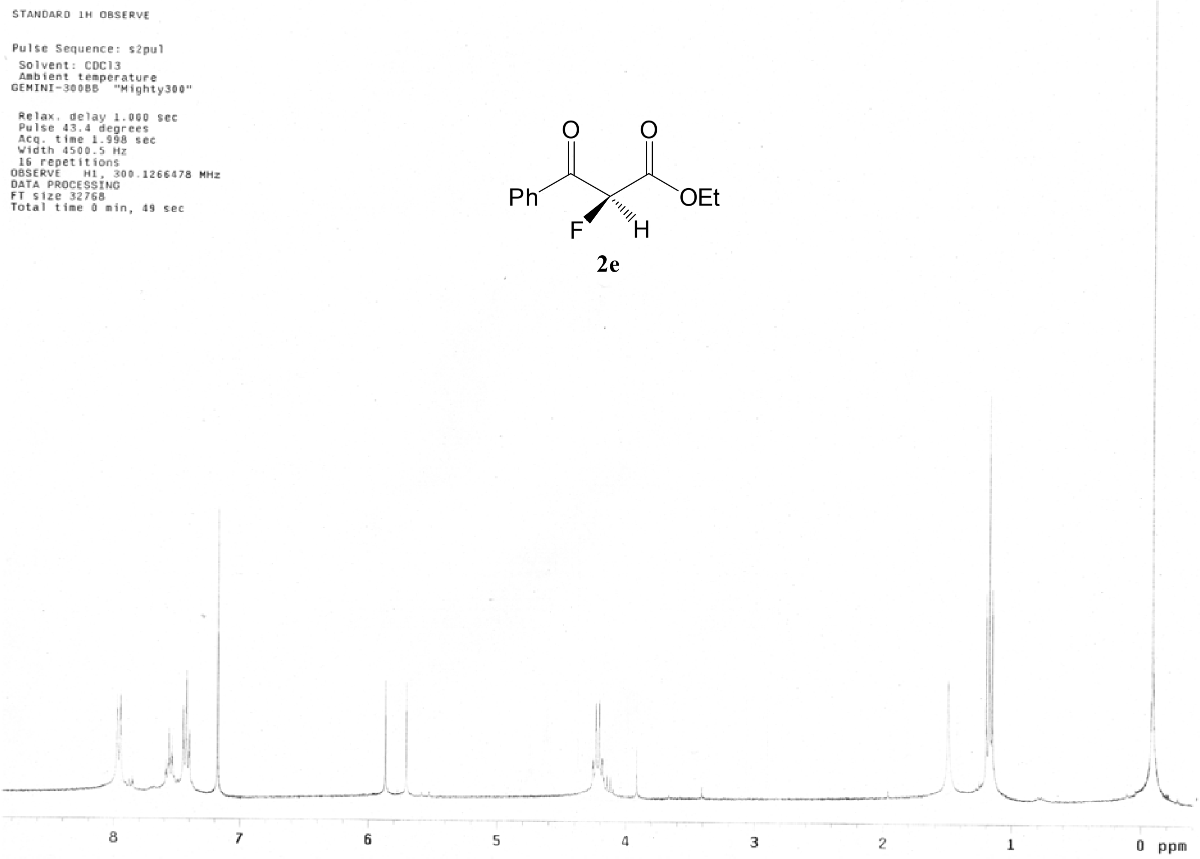
STANDARD 1H OBSERVE
Pulse Sequence: s2pul
Solvent: CDCl3
Ambient temperature
GEMINI-300BB "Mighty300"
Relax. delay 1.000 sec
Pulse 43.4 degrees
Acq. time 1.998 sec
Width 4500.5 Hz
16 repetitions
OBSERVE H1, 300.1266478 MHz
DATA PROCESSING
FT size 32768
Total time 0 min, 49 sec
Ph
OEt
F
H
8
7
6
5
4
3
2
1
0 ppm

2e

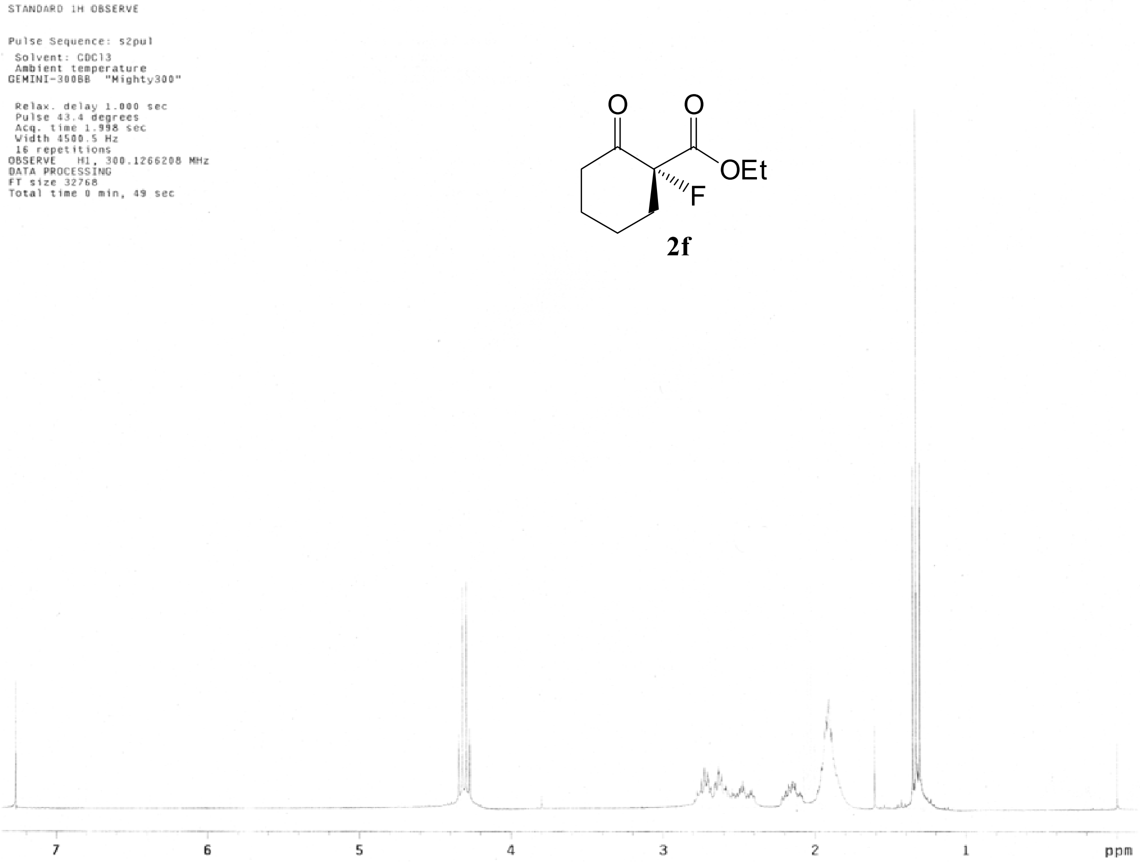
STANDARD 1H OBSERVE
Pulse Sequence: s2pul
Solvent: CDCl3
Ambient temperature
GEMINI-300BB "Mighty300"
Relax. delay 1.000 sec
Pulse 43.4 degrees
Acq. time 1.998 sec
Width 4500.5 Hz
16 repetitions
OBSERVE H1, 300.1266208 MHz
DATA PROCESSING
FT size 32768
Total time 0 min, 49 sec
OEt
F
7
6
5
4
3
2
1
ppm

2f

STANDARD 1H OBSERVE

Pulse Sequence: s2pul
Solvent: CDCl3
Ambient temperature
GEMINI-300BB "Mighty300"

Relax. delay 1.000 sec
Pulse 43.4 degrees
Acq. time 1.998 sec
Width 4500.5 Hz
16 repetitions
OBSERVE H1, 300.1266230 MHz
DATA PROCESSING
FT size 32768
Total time 0 min, 49 sec

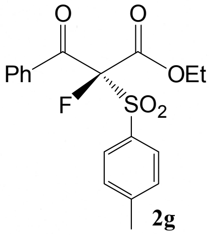

2g

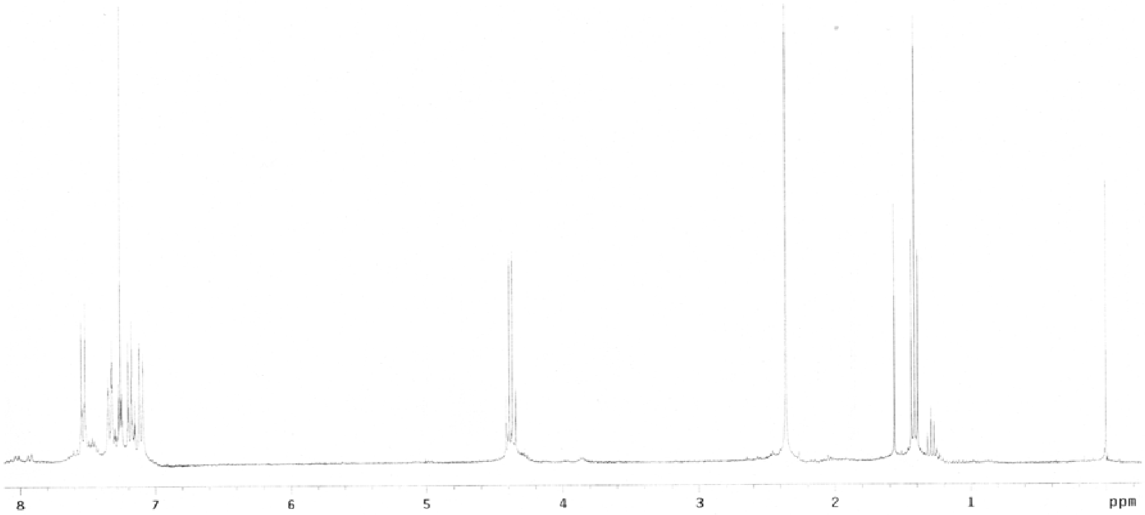

STANDARD 1H OBSERVE

Pulse Sequence: s2pul
Solvent: CDCl3
Ambient temperature
GEMINI-300BB "Mighty300"

Relax. delay 1.000 sec
Pulse 43.4 degrees
Acq. time 1.998 sec
Width 4500.5 Hz
16 repetitions
OBSERVE H1, 300.1266221 MHz
DATA PROCESSING
FT size 32768
Total time 0 min, 49 sec

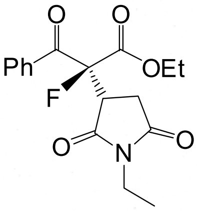

2h

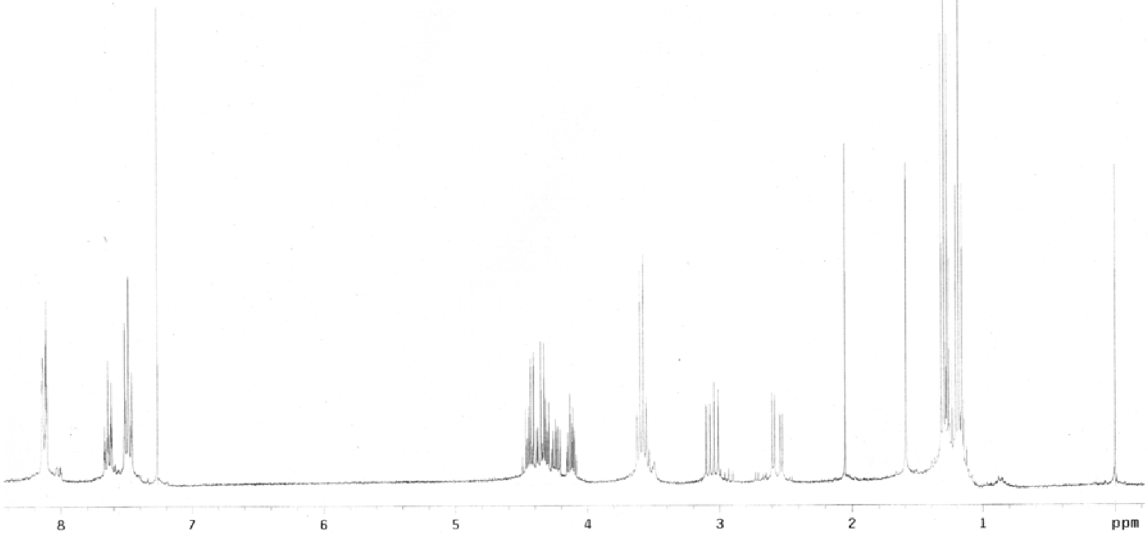

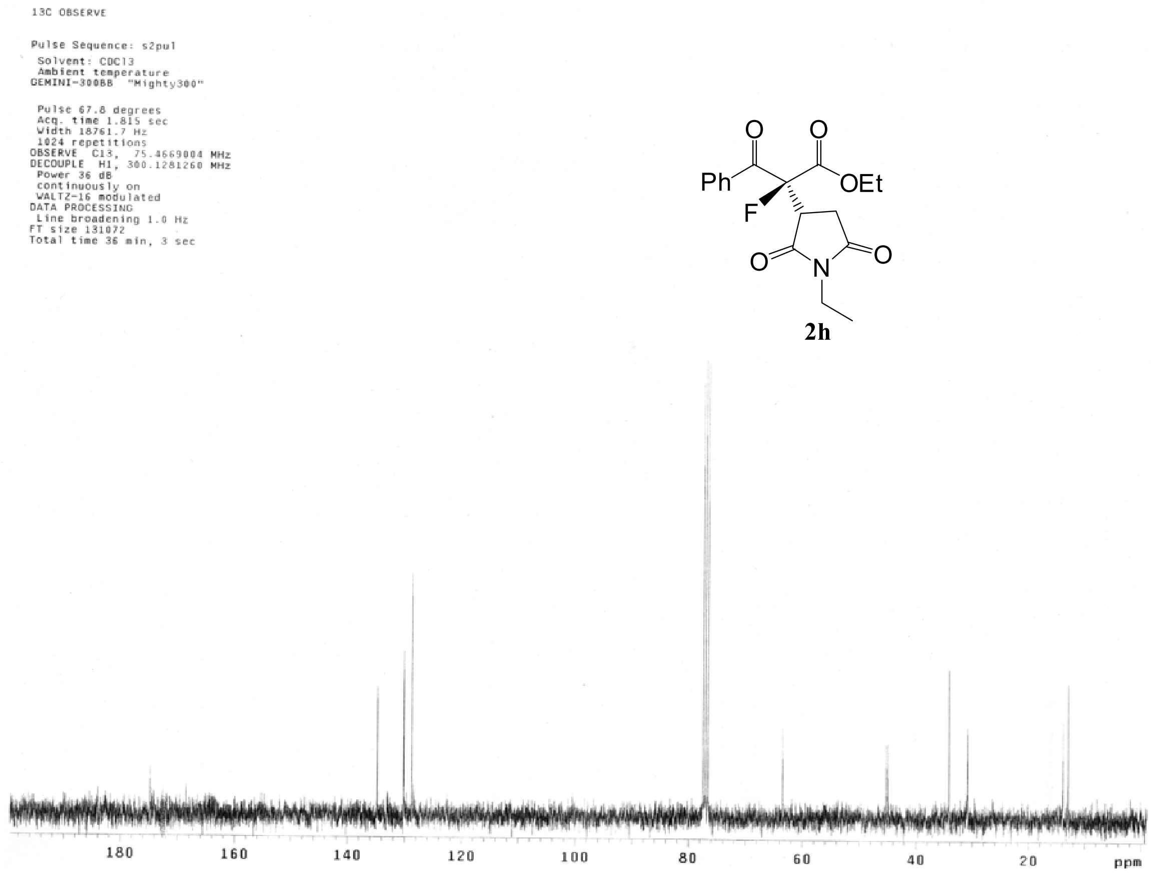
13C OBSERVE
Pulse Sequence: s2pul
Solvent: CDCl3
Ambient temperature
GEMINI-300BB "Mighty300"
Pulse 67.8 degrees
Acq. time 1.815 sec
Width 18761.7 Hz
1024 repetitions
OBSERVE C13, 75.4669004 MHz
DECOUPLE H1, 300.1281260 MHz
Power 36 dB
continuously on
WALTZ-16 modulated
DATA PROCESSING
Line broadening 1.0 Hz
FT size 131072
Total time 36 min, 3 sec
Ph
O
O
OEt
F
O
N
O
2h
180
160
140
120
100
80
60
40
20
ppm

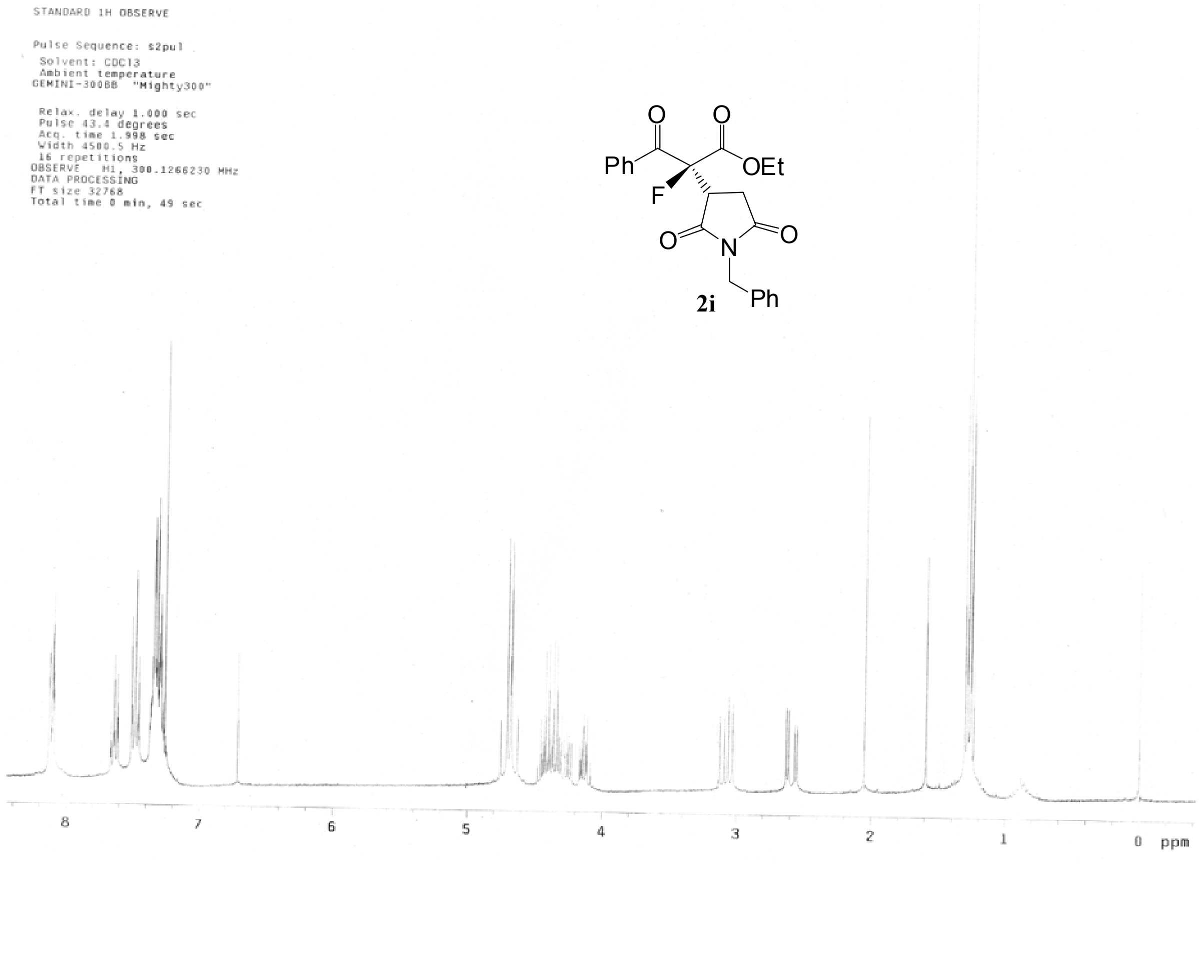
STANDARD 1H OBSERVE
Pulse Sequence: s2pul
Solvent: CDCl3
Ambient temperature
GEMINI-300BB "Mighty300"
Relax. delay 1.000 sec
Pulse 43.4 degrees
Acq. time 1.998 sec
Width 4500.5 Hz
16 repetitions
OBSERVE H1, 300.1266230 MHz
DATA PROCESSING
FT size 32768
Total time 0 min, 49 sec
Ph
O
O
OEt
F
O
N
O
2i
Ph
8
7
6
5
4
3
2
1
0 ppm

13C OBSERVE

Pulse Sequence: s2pul
Solvent: CDCl3
Ambient temperature
GEMINI-300BB "Mighty300"

Pulse 67.8 degrees
Acq. time 1.815 sec
Width 18761.7 Hz
1024 repetitions
OBSERVE C13, 75.4669004 MHz
DECOUPLE H1, 300.1281260 MHz
Power 36 dB
continuously on
WALTZ-16 modulated
DATA PROCESSING
Line broadening 1.0 Hz
FT size 131072
Total time 36 min, 3 sec

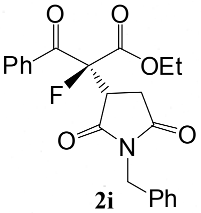

2i

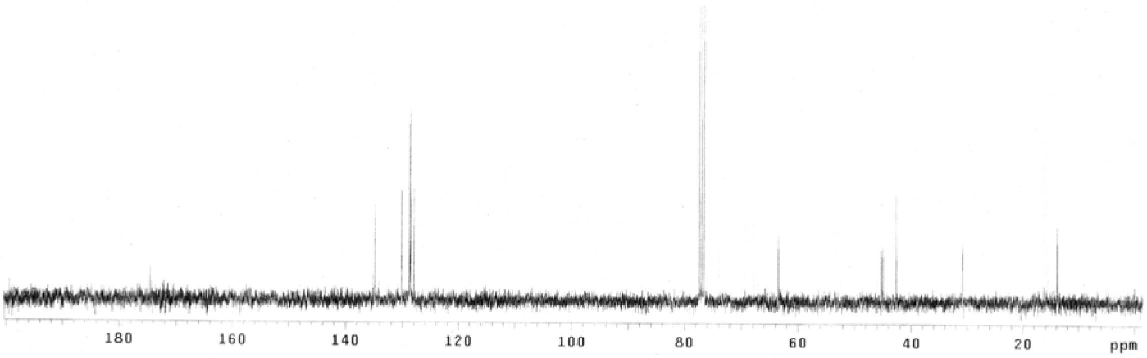

Supplement: File 1 — Chiral HPLC chromatograms for fluorination products 2a–i. LC–MS, NMR spectra for fluorination products 2a–i and cinchona alkaloid derivatives C-1, C-2, C-3 and C-6. LC–MS spectra for 2h and HRMS spectra for C-1. [file Beilstein_J_Org_Chem-08-1233-s001.pdf]
